# Electrocatalytic Reduction of CO_2_ to CO by Molecular Cobalt–Polypyridine Diamine Complexes

**DOI:** 10.3390/molecules29081694

**Published:** 2024-04-09

**Authors:** Yong Yang, Fang Xie, Jiahui Chen, Si Qiu, Na Qiang, Ming Lu, Zhongli Peng, Jing Yang, Guocong Liu

**Affiliations:** 1 School of Chemistry and Materials Engineering, Huizhou University, Huizhou 516001, China; xiefang4498@126.com (F.X.); cc15363451419@163.com (J.C.); qiusi0206@gmail.com (S.Q.); qiangna93@163.com (N.Q.); reggi_lu@hzu.edu.cn (M.L.); zsupzl@126.com (Z.P.); gcl_109@hzu.edu.cn (G.L.); 2 College of Health Science and Environmental Engineering, Shenzhen Technology University, Shenzhen 518118, China

**Keywords:** cobalt, molecular catalyst, electrocatalysis, CO_2_ reduction

## Abstract

Cobalt complexes have previously been reported to exhibit high faradaic efficiency in reducing CO_2_ to CO. Herein, we synthesized capsule-like cobalt–polypyridine diamine complexes [Co(L^1^)](BF_4_)_2_ (**1**) and [Co(L^2^) (CH_3_CN)](BF_4_)_2_ (**2**) as catalysts for the electrocatalytic reduction of CO_2_. Under catalytic conditions, complexes **1** and **2** demonstrated the electrocatalytic reduction of CO_2_ to CO in the presence or absence of CH_3_OH as a proton source. Experimental and computational studies revealed that complexes **1** and **2** undergo two consecutive reversible one-electron reductions on the cobalt core, followed by the addition of CO_2_ to form a metallocarboxylate intermediate [Co^II^(L)–CO_2_^2−^]^0^. This crucial reaction intermediate, which governs the catalytic cycle, was successfully detected using high resolution mass spectrometry (HRMS). In situ Fourier-transform infrared spectrometer (FTIR) analysis showed that methanol can enhance the rate of carbon–oxygen bond cleavage of the metallocarboxylate intermediate. DFT studies on [Co^II^(L)–CO_2_^2−^]^0^ have suggested that the doubly reduced species attacks CO_2_ on the C atom through the d_z_^2^ orbital, while the interaction with CO_2_ is further stabilized by the π interaction between the metal d_xz_ or d_xz_ orbital with p orbitals on the O atoms. Further reductions generate a metal carbonyl intermediate [Co^I^(L)–CO]^+^, which ultimately releases CO.

## 1. Introduction

The transformation of carbon dioxide (CO_2_) to fuels and commodity chemicals is one of the most attractive methods to satisfy the energy demand and decrease atmospheric CO_2_ levels [1,2]. Electrochemical reduction of CO_2_ provides a sustainable approach to achieve this goal [3,4,5,6]. However, the electrochemical conversion of CO_2_ often faces challenges due to the inherent stability of CO_2_, which hinders efficient implementation [7,8]. In nature, the CO-dehydrogenase (CODH) enzyme, containing multi-metallic (Ni, Fe) active sites, is able to convert CO_2_ equivalents into CO efficiently [9]. In the active site of CODH, there is a [Ni-4Fe-5S] cluster and the Ni center is the redox active center to which CO_2_ binds. Therefore, numerous studies have focused on developing active catalysts that mimic the function of the Ni and Fe centers in CODH to promote the electrochemical reduction of CO_2_ [10]. Early studies have focused on low-valent rare metal catalysts (e.g., Re [11,12], Ru [13,14], Ir [15], Rh [16,17]) and have been more recently extended to earth-abundant transition metal catalysts (e.g., Mn [18,19,20,21,22], Fe [8,23,24,25], Ni [26,27], Co [28,29,30,31,32,33]). Among those developments, the cobalt complexes, coordinated with ligands such as porphyrin, cyclam, bipyridyl-N-heterocyclic carbene, polypyridines, and aminopyridines, have exhibited promising results in CO_2_ reduction, owing to their abundant redox performance and the configurational flexibility provided by these ligands [4,6,34].

Cobalt–porphyrin catalysts have been identified as effective catalysts [28,35,36,37]. In aqueous buffered solutions, catalytic reduction of CO_2_ with CoTCPP (TCPP = mesotetracarboxyphenylporphine) leads to the formation of formic acid [36]. Catalytic activity has also been observed with other cobalt–porphyrin catalysts, but the products were not identified [28,35,37]. Several other tetraaza-macrocycles related to cobalt cyclams have shown promise as CO_2_ reduction catalyst [4,6]. Gangi et al. reported that a cobalt complex with the macrocycle 5,7,7,12,14,14-hexamethyl-1,4,8,11-tetraazacyclotetradeca-4,11-diene exhibits a strong positive shift in the Co^ll/l^ reduction potential in the presence of CO_2_ and has an equilibrium constant of 7 × 10^4^ for CO_2_ binding [38]. Cobalt aminopyridyl macrocycle catalysts have also exhibited an advantageous performance as a pivotal constituent of cobalt complexes [4,6]. The cobalt complex [Co^III^N_4_H(Br)_2_]^+^ (N_4_H = 2,12-dimethyl-3,7,11,17-tetraazabicyclo-[11.3.1]-heptadeca-1(7),2,11,13,15-pentaene), which is known to be competent for electrocatalytic H_2_ evolution, with a redox active pyridyldiimine moiety could electro-catalyze CO_2_ to CO in CO_2_-saturated solutions of MeCN with 10 M H_2_O (F_CO_ = 45% ± 6.4) [29]. Jurss et al. showed a series of cobalt catalysts bearing redox-active ligands, based on bipyridyl-N-heterocyclic carbene donors, exhibiting an excellent efficiency for reducing CO_2_ to CO [30]. Marinescu et al. synthesized several macrocyclic catalysts based on azacalix[4](2,6)pyridines. The results indicate that the presence of the pendant NH moiety of the secondary groups leads to a positive shift in the reduction potential of the Co^I/0^ couple, and therefore decreases the overpotential for CO_2_ reduction [31,32,39,40]. Cobalt–polypyridine catalysts are also highly active catalysts [33,41]. Quarter-pyridine [Co(qpy)(H_2_O)_2_]^2+^(qpy = 2,2′:6′,2″:6″,2′′′-quaterpyridine) complex proved to be an active catalyst for CO_2_ to CO (F_CO_ = 90%) reduction at low overpotential of 140 mV in acetonitrile solution with the presence of 3 M phenol [33].

**Scheme 1:**
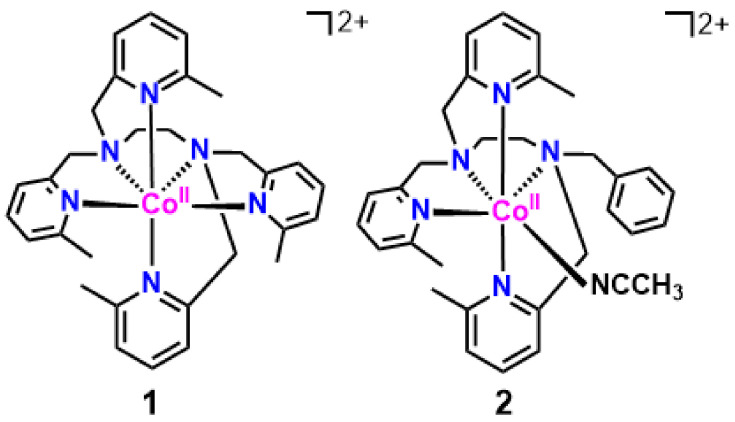
The structures of the target cobalt complexes **1** and **2**.

Though different ligands are involved, one of the key steps in a catalytic CO_2_ reduction is the coordination of CO_2_ molecules to the active site by the reduction catalyst. Herein, we selected capsule-like cobalt(II)–polypyridine diamine complexes [Co(L^1^)](BF_4_)_2_ (**1**; L^1^ = N,N,N′,N′-tetrakis((6-methylpyridin-2-yl)methyl)ethane-1,2-diamine) and [Co(L^2^) (CH_3_CN)](BF_4_)_2_ (**2**; L^2^ = N-benzyl-N,N′,N′-tris((6-methylpyridin-2-yl)methyl)ethane-1,2-diamine) (Scheme 1) as target catalysts. Under catalytic conditions, complexes **1** and **2** demonstrated the electrocatalytic reduction of CO_2_ to CO in the presence or absence of CH_3_OH as a proton source. The results of the cyclic voltammograms (CVs) revealed that the doubly reduced intermediates [Co^0^(L^1^)]^0^ and [Co^0^(L^2^)]^0^ were the catalytic active state. Furthermore, we carefully demonstrated the direct reaction between [Co^0^(L^1^)]^0^ or [Co^0^(L^2^)]^0^ and CO_2_ through FTIR, HRMS, and DFT studies. The results demonstrate that the metallocarboxylate intermediate [Co^II^(L)–CO_2_^2−^]^0^ was successfully detected. Additionally, it is observed that methanol as proton source could accelerate the rate of carbon–oxygen bond breaking of the metallocarboxylate intermediate. Furthermore, it is revealed that the catalytic cycle is mediated by the cobalt core and involves two crucial intermediates, namely [Co^II^(L^1^)–CO_2_^2−^]^0^ and [Co^I^(L^1^)–CO]^+^.

## 2. Results and Discussion

### 2.1. Synthesis, Characterization, and X-ray Structures

The mononuclear cobalt complex [Co(L^1^)](BF_4_)_2_ (**1**) was conveniently prepared by reacting Co(BF_4_)_2_·6H_2_O with L^1^ in acetonitrile at room temperature under ambient air conditions (see the experiment section). A mauve crystalline solid of complex **1** was obtained with a yield of 85%. Complex **2** was synthesized and purified using the same method, yielding 82%. The high-resolution mass spectra (HRMS) and elemental analysis data of complexes **1** and **2** are consistent with the proposed composition of [Co(L^1^)](BF_4_)_2_ and [Co(L^2^)(CH_3_CN)](BF_4_)_2_.

**Figure 1:**
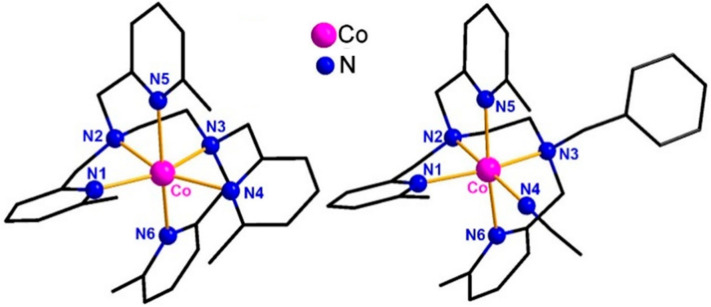
Molecular structures of complexes **1** and **2**. Counter ions and hydrogen atoms are omitted for clarity.

The molecular structures of complexes **1** and **2** were determined by single crystal X-ray crystallography (Figure 1). Complex **1** adopts a distorted octahedral geometry. The Co–N bond distances for two axial pyridyl–nitrogen atoms (Co–N5 (N6) = 2.292 (2.326) Å) are significantly longer than that of the other four nitrogen atoms on the equatorial plane (Co–N1 (N2, N3, N4) = 2.193 (2.164, 2.147, 2.186) Å). Two pyridine rings on the equatorial plane in complex **1** form an angle of extrusion of 34°. By replacing one of the 6-methylpyridinyls in **1** with phenyl, complex **2** features one CH_3_CN solvent molecule coordinated in the equatorial site. Complex **2** shows a normal octahedral geometry, however, its bond lengths and angles are very similar to those in complex **1**. Detailed crystallographic data and processing parameters are collected in Tables S1 and S2.

### 2.2. Electrochemistry

**Figure 2:**
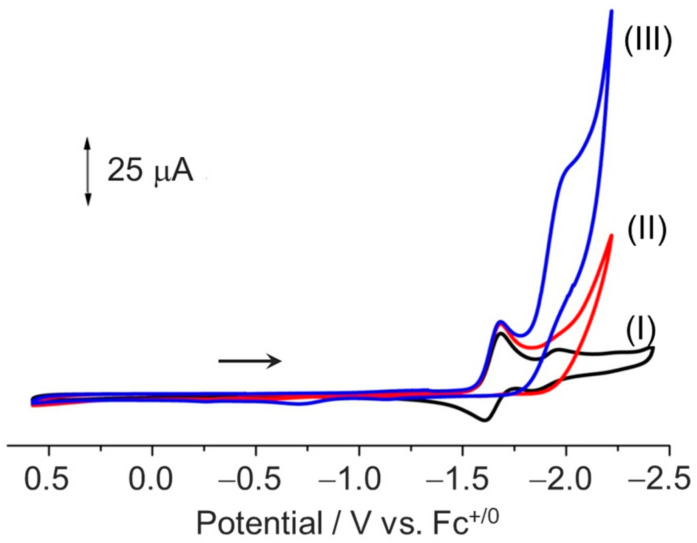
Cyclic voltammograms of complex **1** in CH_3_CN with 0.1 M nBu_4_NPF_6_ as the supporting electrolyte (I) under Ar; under CO_2_ (II) in the absence of and (III) in the presence of 4 M methanol. The arrow in the CV diagram represents the initial scan direction.

Cyclic voltammograms (CVs) of complex **1** under Ar in CH_3_CN solution feature two reversible couples at −1.66 and −1.89 V (all potentials given in this paper are versus ferrocene (Fc^+/0^)) (Figure 2, black line). The free ligand L^1^ is a silent electrochemical in the potential range (Figure S1). Additionally, the absence of any redox processes for a Cu analogue [Cu(L^1^)][BF_4_]_2_ between the potentials of −1.66 to −1.89 V (Figure S2) confirms that the ligand is not responsible for the redox events observed for complex **1**. The results reveal that the two couples can be assigned to Co^II/I^ and Co^I/0^, respectively. The CV of complex **2** (Figure S3) shows almost the same redox couples as **1**. Under the same test conditions, linear sweep voltammograms (LSV) of both complexes **1** and **2** also performed the same redox couples as the reduction waves in the CVs (Figures S4 and S5). Both the first and second couples of complexes **1** and **2** show a good linear relationship between the peak current and squared root of the scan rate, but a difference in slope, indicating that complexes **1** and **2** are functioning in a diffusion-controlled regime and are molecular in nature (Figures S6 and S7). As the scan rate decreases, the ratio of the second peak current to the first increases. Differential pulse voltammograms (DPVs) of complexes **1** and **2** both exhibit two reduction peaks around −1.64 and −1.85 V (Figure S8), which are in accordance with the reduction waves in CVs. Differences in amplitude between Co^II/I^ and Co^I/0^ for both complexes **1** and **2** in the CVs and DPVs may have resulted from minor changes in configuration following the first electron transfer process of Co^II/I^.

Under CO_2_, complex **1** exhibits a slight current enhancement around −1.85 V, which is near the potential for Co^I/0^ couple (Figure 2, red line; Figure S4). A controlled potential electrolysis (CPE) experiment of complex **1** was performed at −1.9 V under CO_2_. Thereafter, an analysis of the gas mixture, in the headspace of the working compartment of the electrolysis cell, by gas chromatography (GC) identified CO as the exclusive gaseous product under the test condition (Figure S9). The catalytic peak current (i_cat_) varies linearly with the concentration of complex **1**, consistent with a mechanism for catalytic CO_2_ reduction that is a first-order reaction in complex **1** (Figure S10). Under CO_2_ in the presence of 4 M methanol, the current exhibits a 4.8-fold increase under CO_2_ conditions in the absence of methanol at −2.1 V (Figure 2 and Figure S11). As documented in the literature, this phenomenon is ascribed to methanol’s role as a proton source, leading to the modification of the catalytic reaction pathway, thereby enhancing the rate of carbon–oxygen bond cleavage of the metallocarboxylate intermediate [4,6,41] (See details in the “The Analysis of Process and Intermediates”). In addition, the catalytic peak current has linear dependence on methanol concentration, ranging from 0.15 to 0.98 M, and reaching saturation at around 4 M (Figure S11). Changing the sequence of bubbling with CO_2_ and adding methanol results in negligible differences in CVs. In contrast, the addition of methanol results in a 260 mV negative shift of the second couple under Ar (Figure 2 and Figure S12). This observation indicates that CO_2_ is much more competitive than methanol to coordinate with the reduced catalyst. Under the same test conditions, complex **2** performs an almost identical phenomenon with complex **1** (Figures S5 and S13–S17).

### 2.3. The Catalytic Rate and Stability of Cobalt Complexes

Under slower scan rates (0.01 to 0.075 V s^−1^) with 4 M MeOH, the CVs of complexes **1** and **2** show a steady “S-shaped” wave (Figures S18 and S19). Higher scan rates lead to the combination of catalytic current plateaus, followed by subsequent increases in current. Using methods described by Savéant [42,43], the turnover frequency (TOF) values for complexes **1** and **2** are estimated to be 2.03 and 1.57 s^−1^, respectively, under the scan rate at 0.05 V s^−1^, with an overpotential around 550 mV (Figures S18 and S19).

A CPE experiment of complex **1** at −2.1 V under CO_2_ shows the CO concentration increases as time goes on (Figure S20 inset). The gas chromatography (GC) analysis confirms that the measured CO production matches well with the theoretical yield (Figure S20), indicating that the faradaic efficiency is close to 100%. However, decomposition of the catalyst under highly reductive conditions results in the CO production deviating from its theoretical yield after 3 h (Figures S20–S22). An average turn-over number (TON) of 9.5 was achieved by complex **1**. Between the changes in complex **1** and **2**, an insignificant difference in catalytic activity and stability was found (Figures S23–S25 and Table S4).

In the presence of methanol under CO_2_ atmosphere, an 80 min CPE experiment of complex **1** performed at −2.1 V. GC analysis shows that CO and H_2_ were the main products. The faradaic efficiency of CO is approximately 30% within 40 min and shows a gradually decreasing trend, dropping to 7% at 80 min. The faradaic efficiency of H_2_ gradually increased, reaching 85% at 80 min (Figure S26). Additionally, obvious deposits were observed on the surface of glass carbon electrode after electrolysis for 80 min (Figure S27). This phenomenon is commonly observed in published papers for molecular catalysts, where the predominant component of deposition is a zero-valent metal, accompanied by a minor presence of metal oxide [31,44,45,46]. The chemical state of the deposits on the surface of glass carbon electrode were investigated by XPS. As shown in Figure S28, the peak with binding energies at 778.5 eV are associated with the zero-valent cobalt, and the peak of 779.5 and 781.1 eV binding energies stem from the cobalt in the oxidation state. The area of the three peaks indicates that the zero-valent cobalt is the predominant component in our experimental deposition. The obtained results align with the findings reported in published papers on cobalt molecular catalysts [31]. The deposited glass carbon electrode, acting as the working electrode, was used at −2.1 V in the presence of methanol under CO_2_ atmosphere without complex **1** or **2**. Thereafter, GC analysis shows that H_2_ was the sole product. The results reveal that deposits are incapable of catalyzing the reduction of CO_2_; their catalytic activity is limited to the reduction of protons for hydrogen gas production. The actual catalyst responsible for the conversion of CO_2_ into CO is complexes **1** or **2** in CH_3_CN solution.

### 2.4. The Process and Intermediates Analysis

The initial step in the reduction of CO_2_ molecules involves its binding. As shown in Figure 2, in the presence of CO_2_, [Co^0^(L^1^)]^0^ is sensitive to CO_2_ such that slightly more negative scanning following Co^II/I^ would result in the irreversibility of the Co^I/0^ and Co^II/I^ couple (Figure S29). In the DPV test of complex **1** under CO_2_, the first peak shows no difference with that under Ar, but the second peak expands to a plateau (Figure S30), indicating that CO_2_ binding and subsequent reduction coincide with the Co^I/0^ couple. In the presence of 4 M methanol, the plateau splits into two stronger peaks. The more positive one could be ascribed to the formation of a CO_2_ adduct [Co^II^(L^1^)–COOH^−^]^+^. The other corresponds to the following reduction. Additionally, the reduction of complex **1** is restricted to the [Co^I^(L^1^)]^+^ state by mixing complex **1** with 1 equiv of bis-pentamethylcyclopentadienyl-cobalt(II) (Cp*_2_Co, the oxidation potential of Cp*_2_Co is −1.82 V vs. Fc^+/0^ in CH_3_CN). Then, the addition of dry CO_2_ brings no new absorption band attributed to carbonyl. Herein, it can be concluded that [Co^0^(L^1^)]^0^ is the active state for CO_2_ rather than [Co^I^(L^1^)]^+^, and two–electron reduction are necessary to activate complex **1**.

**Figure 3:**
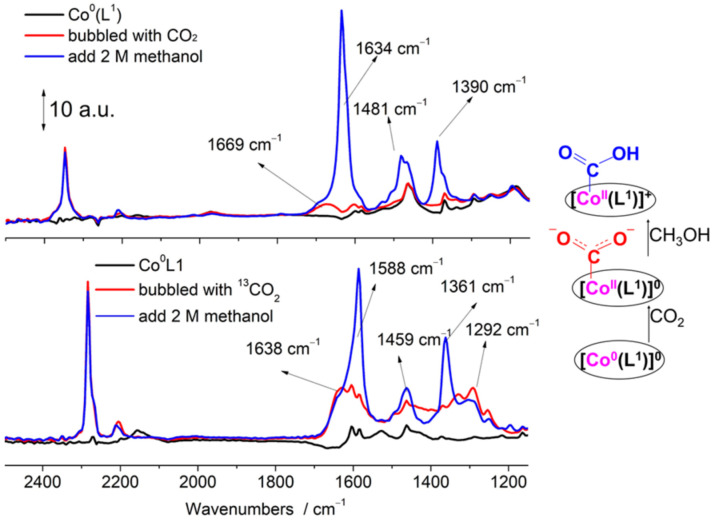
IR spectrum of freshly prepared [Co^0^(L^1^)]^0^ (black line), which is bubbled with dry CO_2_ (above) and ^13^CO_2_ (down) (red line), then 2 M methanol is added (blue line). These lines were recorded by in situ FTIR during the reaction process. The tests are performed in a CH_3_CN/THF (1:1) mixture under Ar or CO_2_ with 10.0 mM [Co^0^(L^1^)]^0^.

[Co^0^(L^1^)]^0^ as a dark blue solution in a mixture of CH_3_CN/THF (1:1) can be easily prepared by chemical reduction of complex **1** with 2 equiv of NaHg. When the solution is bubbled with dry CO_2_ gas, the color changes back to mauve in 30 s (Figure S31). As we know, the blue color is attributed to the electron transfer between d orbitals of cobalt. The significant change in color indicates two electrons are transferring from cobalt to CO_2_. Then, we monitor the process of CO_2_ coordinating with [Co^0^(L^1^)]^0^ by in situ FTIR (Figure 3 and Figure S32). In the range of 1200 to 2500 cm^−1^, [Co^0^(L^1^)]^0^ shows a slight IR absorption. When dry CO_2_ is added, a weak broad absorption appears at 1669 cm^−1^, indicating that CO_2_ coordinates with [Co^0^(L^1^)]^0^ to form the intermediate [Co^II^(L^1^)–CO_2_^2−^] (Figure 3). The frequency is close to that reported for Co(salen)CO_2_Na (1680 cm^−1^) [47]. Then, upon addition of methanol, three new bands appeared at 1634, 1481, and 1390 cm^−1^ (Figure 3 and Figure S33). The assignment was confirmed by isotope labelling experiment. When dry ^13^CO_2_ is introduced, followed by the addition of methanol, these new bands shift toward lower wavenumbers by 30 to 60 cm^−1^ (Figure 3). We conclude that methanol disrupts the balance of electron distribution on the two oxygen atoms of CO_2_, resulting ultimately in coordinated transformation into the metallocarboxylate intermediate (Figure 3). Therefore, in this catalysis system, methanol could lead to the CO_2_ binding equilibrium moving toward the forward reaction.

**Figure 4:**
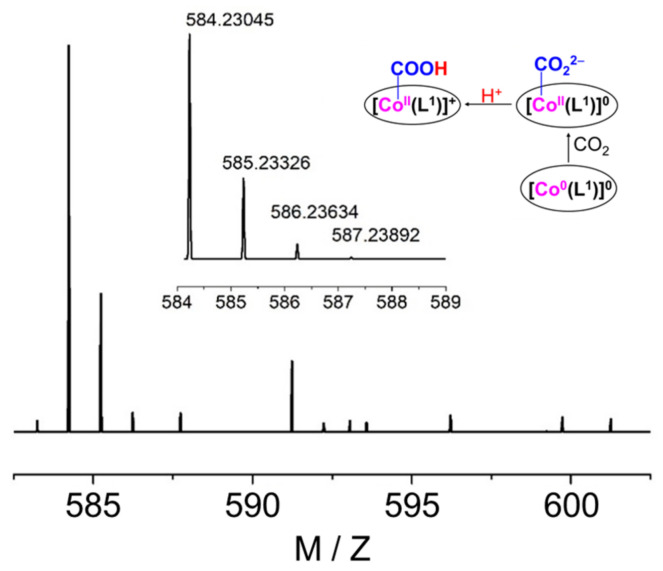
The observed and calculated (inset) HRMS of the protonated product of [Co^II^(L^1^)−CO^2−^]^0^ generated from CO_2_ coordinating to [Co^0^(L^1^)]^0^.

The fresh reaction solution is then analyzed by high resolution mass spectrometry (HRMS) [48]. The isotope distribution pattern at m/z^−^ = 584.2305, 585.2333 and 586.2363 fit well with the calculated isotope distribution pattern of the mass fragment [Co^II^(L^1^)–COOH^−^]^+^. The results reveal that we successfully detected the protonated product of [Co^II^(L^1^)–CO_2_^2−^]^0^ (Figure 4 and Figure S34). In the same way, the protonated product of [Co^II^(L^2^)–CO_2_^2−^]^0^ (Figures S35 and S36), prepared from the analogue complex **2**, was also detected by HRMS and it was found that the acetonitrile ligand had dissociated from cobalt. These results provide clear evidence for the incorporation of CO_2_ into the [Co^0^(L^1^)]^0^ intermediate.

**Figure 5:**
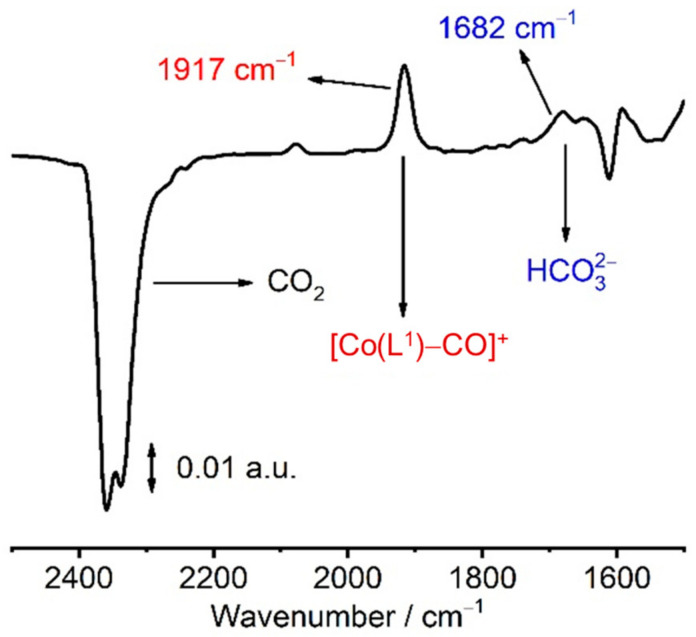
Differential IR-SEC spectrum of complex **1** (10.0 mM) recorded after electrolysis at −1.85 V (vs. Fc^+/0^) for 10 s in CO_2_ saturated CH_3_CN solution.

As referred to in the DPV test of complex **1** under CO_2_, CO_2_ binding and the following reduction coincide with the Co^I/0^ couple (Figure S30). To investigate the subsequent reduction of the CO_2_-coordinated cobalt metallocarboxylate, in situ infrared spectroelectrochemistry (IR-SEC) experiments are performed at −1.85 V. The differential IR-SEC spectrum of **1** reveals a decrease in CO_2_ intensity around 2340 cm^−1^ and the emergence of a band at 1917 cm^−1^ (Figure 5). Complex **1** itself does not possess IR absorption modes with sufficient intensity that can be distinguished within the range of 1500 to 2500 cm^−1^, under varied electrolysis potential from −1.0 to −2.4 V, and in the absence of CO_2_. The absorption peak observed at 1917 cm^−1^ can be ascribed to the two-electron reduction species [Co^I^(L^1^)–CO]^+^ [26,49], which is obtained from through further reduction of [Co^II^(L^1^)–CO_2_^2−^]^0^. Fujita and colleagues have reported that [Co^I^(macrocycle)–CO]^+^ (macrocycle = 5,7,7,12,14,14-hexamethyl-1,4,8,11-tetraazacyclotetradeca-4,11-diene) exhibits an absorption peak at 1916 cm^−1^ [49,50]. Upon reduction of complex **1** with 1 equiv of Cp*_2_Co under CO, a weak IR absorption band resembling [Co^I^(macrocycle)–CO]^+^ was observed at 1917 cm^−1^ (Figure S37). In the presence of 1 M methanol under the same experimental condition, two strong peaks at 1916 and 1664 cm^−1^ were ascribed to [Co^I^(L^1^)–CO]^+^ and the combination absorption of [Co^II^(L^1^)–COOH^−^]^+^, respectively (Figure S38). The differential IR-SEC spectrum of complex **2** was also recorded at −1.85 V. Similar to complex **1**, the peak of the reduction species [Co^I^(L^2^)–CO]^+^ was also observed at 1917 cm^−1^. The findings suggest that complex **2** undergoes the same catalytic mechanism as complex **1**. (Figure S39). Consequently, there will be further reductions of CO_2_-coordinated cobalt intermediates to generate CO.

### 2.5. Density Functional Theory (DFT) Calculation and Mechanistic Consideration

To get a better understanding of the electronic distribution on the reduced catalyst [Co^0^(L^1^)]^0^, we employed DFT calculations using Gaussian 09, Revision D.01. Firstly, the geometry-optimized initial structure of [Co^I^(L^1^)]^+^ shows that both pyridine groups in the axial direction dissociate from cobalt (Figure S40a). The dissociation is consistent with the longer C–N distance in the axial direction. The dissociation results in a minor change in configuration, which leads to the difference in current amplitude for the two reduction peaks in complex **1** (Figure 2). As proven above, CO_2_ coordination is coupled with the second reduction. After which, the CO_2_ molecule is joined to the optimized structure [Co^I^(L^1^)]^+^ at the axial open site. Meanwhile, the second electron is added. The geometry-optimized electronic structure of [Co^II^(L^1^)–CO_2_^2−^]^0^ shows the electron density distribution in the frontiers (Figure S40b). The optimized structure provides evidence supporting the hypothesis that CO_2_ can undergo an attack by the d_z_^2^ orbital of the doubly reduced cobalt from the axial direction. The formation of a σ bond between the d_z_^2^ orbital and the carbon atom of CO_2_ (HOMO) is observed, and this interaction with CO_2_ can be further stabilized through π interactions between the metal’s d_xz_ or d_yz_ orbitals and p orbitals on the oxygen atoms of CO_2_ (HOMO–5, Figure S41). The results of the Kubiak and Fujita groups are also similar in that the stabilizing interaction is with oxygen atoms of CO_2_ rather than the carbon atom [51]. This tridentate interaction makes the coordination of CO_2_ to [Co^0^(L^1^)]^0^ irreversible. Furthermore, the attack on the oxygen atom by Brönsted acid becomes more favorable. The optimized [Co^II^(L^1^)–CO_2_^2−^]^0^ shows that one dissociated pyridine in [Co^I^(L^1^)]^+^ state stabilizes the CO_2_ adduct from the axial direction (Figure S38). Compared with **1**, **2** features one labile CH_3_CN ligand. The reductive dissociation of the labile ligand acetonitrile from the polypyridine–cobalt complexes at the [Co^I^] state has been previously verified by other groups. By replacing acetonitrile with CO_2_, while two electrons are also added, the geometry-optimized electronic structure of [Co^II^(L^2^)–CO_2_^2−^]^0^ gives rise to almost the same electronic structure as [Co^II^(L^1^)–CO_2_^2−^]^0^ (Figures S42 and S43). Thus, we conclude complex **2** to be a simpler model for CO_2_ reduction.

**Scheme 2:**
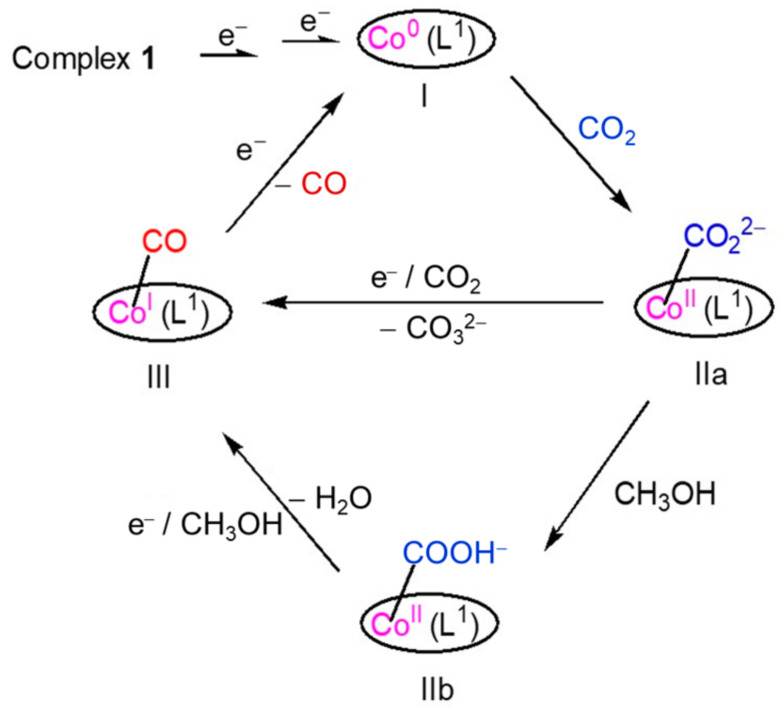
A proposed mechanism for electroreduction of CO_2_ to CO catalyzed by complex **1**.

Taking complex **1** as an example, Scheme 2 summarizes a proposed mechanism for the catalytic reduction of CO_2_ to CO by complexes **1** and **2**. Initially, a two-electron reduction is necessary to generate the active complex [Co^0^(L^1^)]^0^ (**I**). As [Co^0^(L^1^)]^0^ is so sensitive, CO_2_ is coordinated to generate [Co^II^(L^1^)–CO_2_^2−^]^0^ (**IIa**). Subsequently, the carbon–oxygen bond cleavage have two paths: (1) another one-electron reduction that leads to O^2−^ transferring to another CO_2_ molecule, resulting in the intermediate of [Co^I^(L^1^)–CO]^+^ (**III**) and coproduct CO_3_^2−^; or (2) the intermediate of [Co^II^(L^1^)–COOH]^+^ (**IIb**) is formed first in the presence of CH_3_OH as a proton source, and it then receives an electron and undergoes reduction, leading to the cleavage of the carbon–oxygen bond and generating the intermediate [Co^I^(L^1^)–CO]^+^ (**III**) and coproduct H_2_O. Finally, one more electron is required to release CO and regenerate [Co^0^(L^1^)]^0^. The involvement of intermediates [Co^II^(L^1^)–CO_2_^2−^]^0^ (**IIa**) and [Co^I^(L^1^)–CO]^+^ (**III**) indicates that the cobalt core mediates this catalytic process.

## 3. Materials and Methods

### 3.1. Materials and Instruments

All manipulations for the preparation and handling of the organometallic complexes were carried out under air. All the solvents were used as received. Other commercially available chemicals, such as Co(BF_4_)_2_·6H_2_O, 1,2-ethanediamine,6-methylpicolinaldehyde, N-benzylethane-1,2-diamine, were purchased from local suppliers and used as received. Water was deionized with the Millipore Milli-Q UF Plus system. Glass carbon discs (3 mm), AgCl/Ag electrodes and platinum wire were purchased from CHI for electrochemical studies. Arrows are employed in the CV diagram to denote the initial scanning direction.

The NMR spectrum was acquired by employing the Varian INOVA 600 NMR spectrometer, an instrument manufactured by the renowned American company Varian. Mass spectra were recorded with HP 1100 HPL/ESI-DAD-MS and Waters/Micromass LC/Q-TOF-MS instruments. Elemental analyses were performed with a Thermoquest-Flash EA 1112 elemental analyzer. FTIR in situ was monitored by React IR 15 instrument equipped with a MCT detector and a Dsub AgXSiComp in situ probe.

### 3.2. Synthesis

Synthesis of ligands L^1^ and L^2^. L^1^ and L^2^ were prepared according to the procedures in the literature [52,53].

L^1^: ^1^H NMR (CDCl_3_, 400 MHz), *δ* 7.45 (d, 4H), 7.32 (m, 4H), 6.95 (d, 4H), 3.75 (s, 8H), 2.76 (s, 4H), 2.49 (s, 12H).

L^2^: ^1^H NMR (CDCl_3_, 400 MHz), *δ* 7.45 (m, 4H), 7.26 (m, 6H), 6.70 (m, 4H), 3.75 (s, 6H), 3.59 (s, 2H), 2.76 (d, 4H), 2.50 (s, 9H).

Preparation of complexes **1** and **2**. Compound Co(BF_4_)_2_·6H_2_O (1.1 equiv) and L^1^ (1.0 equiv) or L^2^ (1.0 equiv) were added to the CH_3_CN solution (5 mL). The mixture was stirred at room temperature for 12 h. The mauve solution was then transferred to tubes, which were placed in the flask containing ether, and needle-shaped crystals were obtained.

Complex **1**: elemental analysis calcd (%) for C_30_H_36_B_2_CoF_8_N_6_ (%): C, 50.53; H, 5.08; N, 11.77; found: 50.52; H, 5.0.9; N, 11.78; HRMS: m/z = 269.6641 {[M–2(BF_4_)^−^]/2}^+^, 574.3025 [M–2(BF_4_)^−^+Cl^−^]^+^.

Complex **2**: elemental analysis calcd (%) for C_32_H_38_B_2_CoF_8_N_6_ (%): C, 51.98; H, 5.19; N, 11.38 found: C, 52.00; H, 5.19; N, 11.39; HRMS: m/z = 262.1598 {[M–2(BF_4_)^−^–CH_3_CN]/2}^+^, 559.3027 [M–2(BF_4_)^−^–CH_3_CN+Cl^−^]^+^.

In situ preparation of [Co^0^(L^1^)]^0^. Complex **1** was mixed with 2.0 equiv of Na(Hg) in a highly pure THF and CH_3_CN (1:1) mixture under Ar. The sample was stirred under Ar overnight. A dark blue solvent appeared after stirring stopped. The freshly prepared [Co^0^(L^1^)]^0^ was used in the next step without further handling.

In situ preparation of [Co^II^(L^1^)–CO_2_^2−^]^0^. The reaction was monitored by a React IR 15 instrument equipped with a MCT detector and a Dsub AgXSiComp in situ probe. The experiment was performed at room temperature. [Co^II^(L^1^)–CO_2_^2−^]^0^ was prepared by bubbling dry CO_2_ into the solution of complex [Co^0^(L^1^)]^0^. In about 30 s, the dark blue color changed back to pink. After which, 2 M of CH_3_OH was added into the solvent. The fresh reaction solution was tested directly by a mass spectrum.

In situ preparation of [Co^II^(L^1^)–CO]^2+^(BF_4_)_2_ and [Co^I^(L^1^)–CO]^+^(BF_4_). The reaction was monitored by a React IR 15 instrument equipped with a MCT detector and a Dsub AgXSiComp in situ probe. The experiment was performed at room temperature. [Co^II^(L^1^)–CO]^2+^(BF_4_)_2_ was prepared by bubbling CO into the solution of complex [Co(L^1^)](BF_4_)_2_(**1**) in acetonitrile. In about 3 min, the absorption attributed to [Co^II^(L^1^)–CO]^2+^(BF_4_)_2_ reached the highest intensity. [Co(L^1^)–CO]^+^(BF_4_)^−^ was prepared by the addition of 1 equiv Cp*_2_Co (Bis-pentamethylcyclopentadienyl-cobalt(II)) into the solution of complex [Co(L^1^)](BF_4_)_2_ in acetonitrile under CO atmosphere. In about 3 min, the absorption attributed to [Co(L^1^)–CO]^+^(BF_4_)^−^ reached the highest intensity.

### 3.3. IR-SEC Test Details

The spectroelectrochemical experiments were conducted using an IFS 66v/S FTIR spectrometer (Bruker) equipped with an MCT-A detector. Electrode potential was controlled using a model CHI potentiostat. The SEC experiments were performed in a homemade three-neck cell with a glassy carbon electrode serving as the working electrode, while a platinum foil acted as the counter electrode, and Ag/AgCl (3 M KCl) as the reference electrode. IR-SEC spectra were collected in a single beam mode at either 2 cm^−1^ or 4 cm^−1^, and differential absorption spectra were presented relative to the reference spectrum and were recorded immediately prior to applying the potential.

### 3.4. Computational Methods

All DFT computations were calculated at the B3LYP (the Becke three-parameter Lee–Yang–Parr hybrid functional) level using the 6-31G(d) basis set for all atoms via the Gaussian 09 program package [54,55]. All structures were fully optimized and confirmed as minima by analytical vibrational frequency calculations with B3LYP/6-31G(d). The solvent acetonitrile was modeled as a dielectric continuum using a polarizable continuum model (PCM). The basis sets and solvent model were adopted to predict CO coordinates and the results were in good agreement with X-ray crystal structure [56]. All calculated structures were visualized by Gauss View 5.0.9.

## 4. Conclusions

In conclusion, this paper provides a comprehensive investigation into the electrocatalytic reduction of CO_2_ to CO mediated by the capsule-like cobalt(II)–polypyridine diamine complexes **1** and **2**. The catalytically active state is represented by [Co^0^(L^1^)]^0^ or [Co^0^(L^2^)]^0^. The catalytic cycle involves crucial intermediates, namely [Co^II^(L)–CO_2_^2−^]^0^ and [Co^I^(L)–CO]^+^, with the cobalt core playing a central role. These findings provide comprehensive mechanistic insights into a class of catalysts enabling the sustained electrocatalytic reduction of CO_2_ to CO, emphasizing the crucial role of prior reductions on cobalt alone. These discoveries have guided us in synthesizing a series of cobalt-based complexes with secondary coordination spheres (such as hydroxyl, carboxyl, and amino groups) to facilitate the cleavage of carbon–oxygen bonds in intermediates, thereby enhancing the electrocatalytic performance for the CO_2_ reduction exhibited by these cobalt-based complexes.

## Data Availability

Data are contained within the article and Supplementary Materials.

## Supplementary Materials

The following supporting information can be downloaded at: https://www.mdpi.com/article/10.3390/molecules29081694/s1.
