# Supplementary material for: Electrocatalytic Reduction of CO2 to CO by Molecular Cobalt–Polypyridine Diamine Complexes
Source: Molecules. 2024 Apr 9;29(8):1694. doi: 10.3390/molecules29081694 (PMC11051790; doi:10.3390/molecules29081694)
Supplement: Supplementary file 1 [file molecules-29-01694-s001.zip › molecules-2930820-supplementary.pdf]

Supporting Information for

Electrocatalytic Reduction of CO_2 to CO by Molecular Cobalt Poly-Pyridine-Diamine Complexes

Yong Yang,[*a] Fang Xie,[a] Jiahui Chen,[a] Si Qiu,[a] Na Qiang,[a] Ming Lu,[a] Zhongli Peng,[a] Jing Yang,[*b] and Guocong Liu,[*a]

a School of Chemistry and Materials Engineering, Huizhou University, Huizhou, Guangdong 516001, China.

b College of Health Science and Environmental Engineering, Shenzhen Technology University, Shenzhen 518118, China.

Corresponding Authors
Email*: yangyongself@hzu.edu.cn
Email*: yangjing2@sztu.edu.cn
Email*: gcl_109@163.com

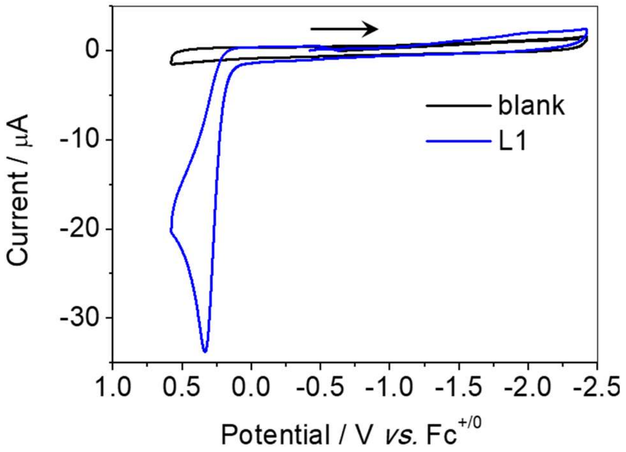

Figure S1. Cyclic voltammogram of ligand L[1] (1.0 mM) in $^{n}Bu_4NPF_6$ acetonitrile solution (0.1 M) with a scan rate of 100 mV/s.

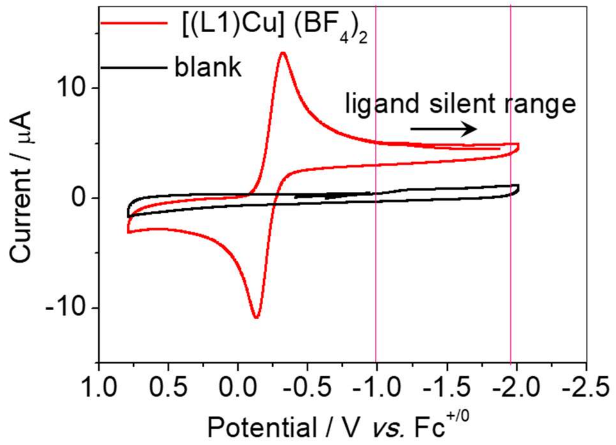

Figure S2. Cyclic voltammogram of complex [Cu (L[1])]$(BF_4)_2$ (1.0 mM) in $^{n}Bu_4NPF_6$ acetonitrile solution (0.1 M) with a scan rate of 100 mV/s.

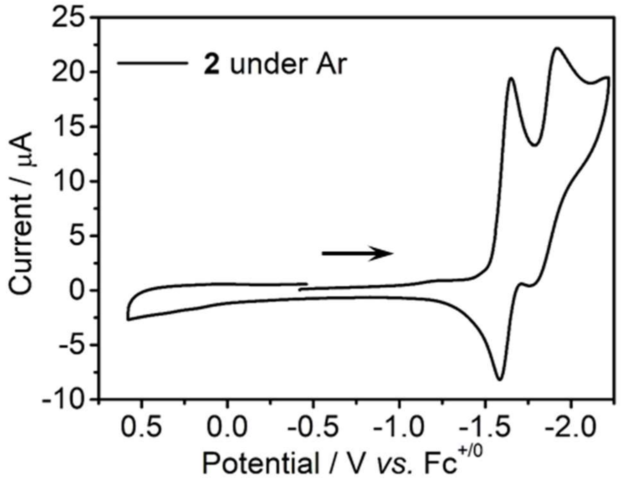

Figure S3. Cyclic voltammograms of complex **2** (1.0 mM) in $^{n}Bu_4NPF_6$ acetonitrile solution (0.1 M) with a scan rate of 100 mV/s.

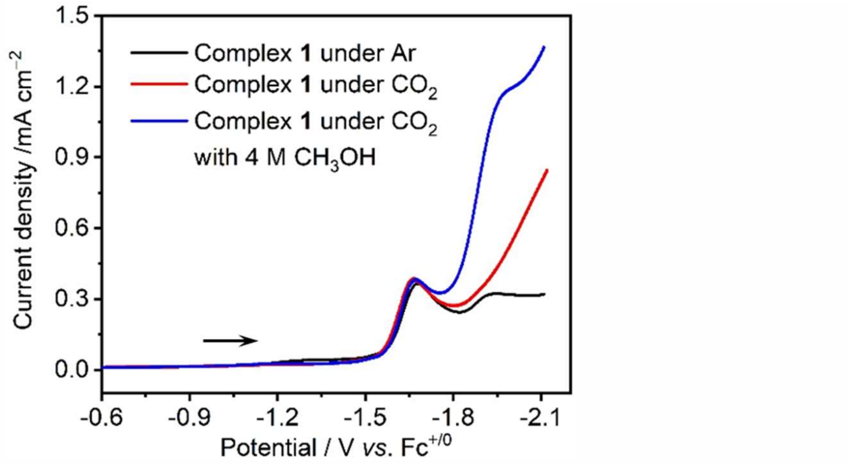

Figure S4. LSV curves of complex **1** (1.0 mM) in acetonitrile solution (0.1 M ^{n}Bu$_4$NPF$_6$) with a scan rate of 100 mV/s.

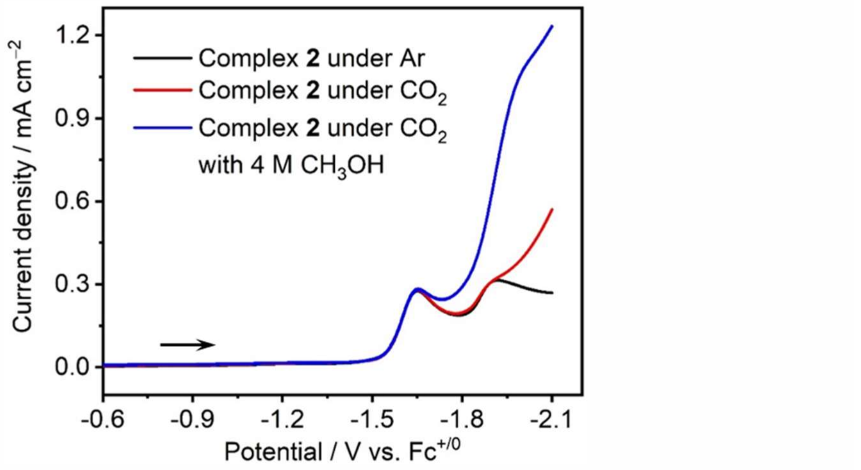

Figure S5. LSV curves of complex **2** (1.0 mM) in acetonitrile solution (0.1 M ^{n}Bu$_4$NPF$_6$) with a scan rate of 100 mV/s.

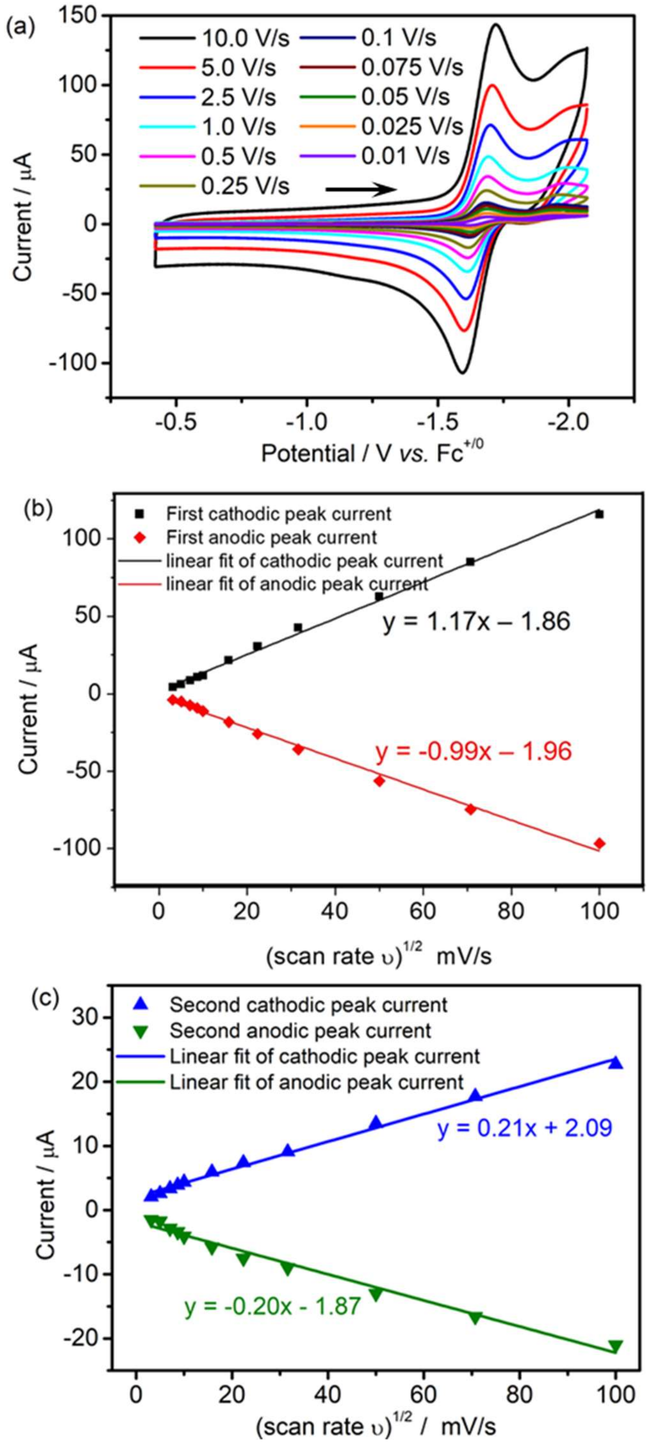

Figure S6. (a) The $Co^{II/I}$ and $Co^{I/0}$ couple of complex **1** (1 mM) in $^{n}Bu_4NPF_6$ (0.1 M) acetonitrile solution with scan rate varied from 0.01 V/s to 10 V/s under Ar; (b) Plot of cathodic and anodic peak current (i_p) vs. $v^{1/2}$ for the first redox couple of complex **1**. (c) Plot of cathodic and anodic peak current (i_p) vs. $v^{1/2}$ for the second redox couple of complex **1**.

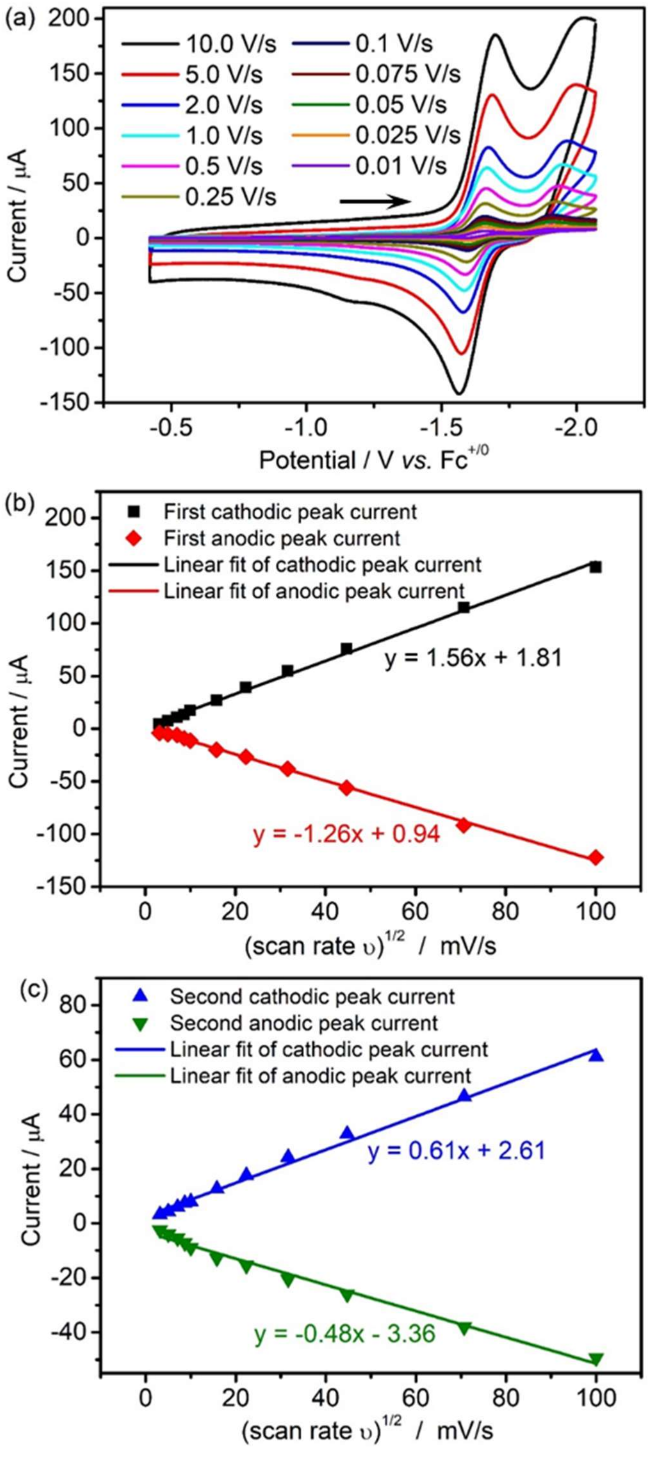

Figure S7. (a) The $Co^{II/I}$ and $Co^{I/0}$ couple of complex **2** (1 mM) in $^{n}Bu_4NPF_6$ (0.1 M) acetonitrile solution with scan rate varied from 0.01 V/s to 10 V/s under Ar; (b) Plot of cathodic and anodic peak current (i_p) vs. $v^{1/2}$ for the first redox couple of complex **2**. (c) Plot of cathodic and anodic peak current (i_p) vs. $v^{1/2}$ for the second redox couple of complex **2**.

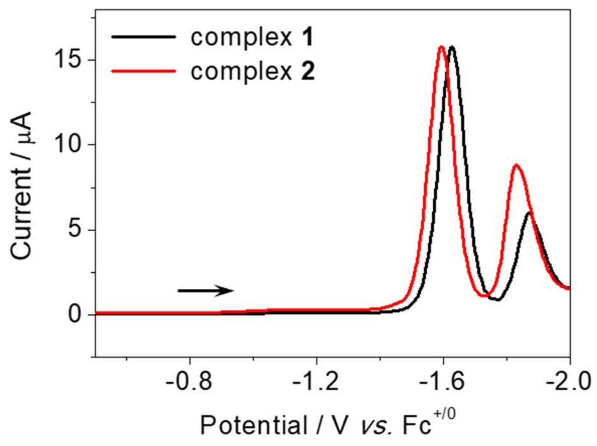

Figure S8. Differential pulse voltammograms of complexes **1** and **2** (1.0 mM) in $^{n}Bu_4NPF_6$ acetonitrile solution (0.1 M) at a scan rate of 4 mV s^{-1}.

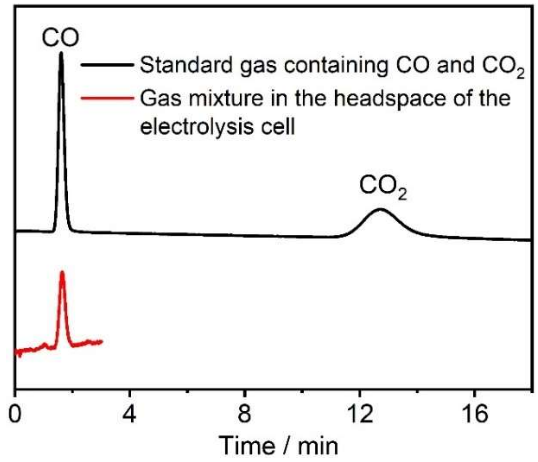

Figure S9. Gas chromatography analysis of the standard gas containing CO and CO_2 (black line) and gas mixture in the headspace of the electrolysis cell (red line, controlled potential electrolysis of complex **1** in acetonitrile solution under CO_2 at –1.9 V for half an hour).

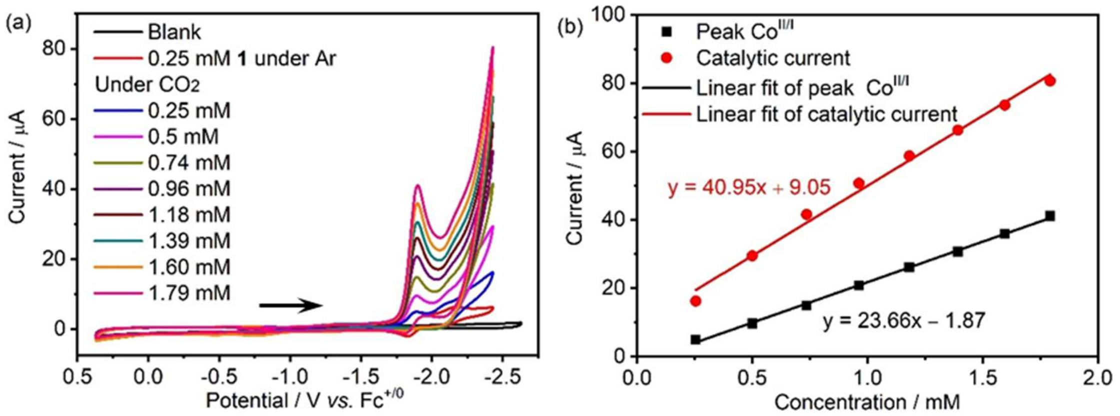

Figure S10. (a) Cyclic voltammograms of complex **1** in CO_2 saturated acetonitrile solution with **1** concentration varied from 0.25 to 1.79 mM at a scan rate of 100 mV s^{-1}. (b) Plot of the reduction current maximum of $Co^{II/I}$ couple and catalytic current maximum as a function of catalyst concentration.

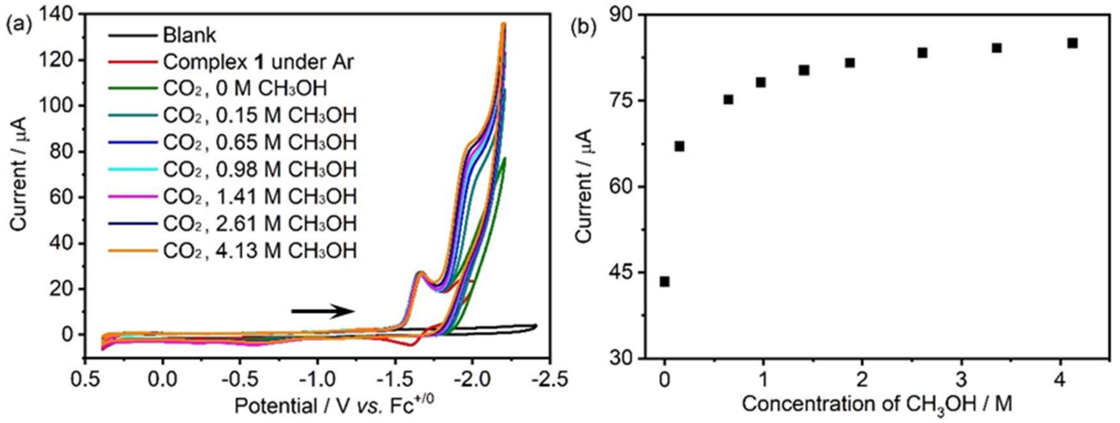

Figure S11. (a) Cyclic voltammogram of complex **1** (1.0 mM) in CH_3CN solution with the presence of varying concentration of CH_3OH at a scan rate of 100 mV/s under CO_2; (b) The trend of catalytic peak current with increasing methanol concentration at −2 V.

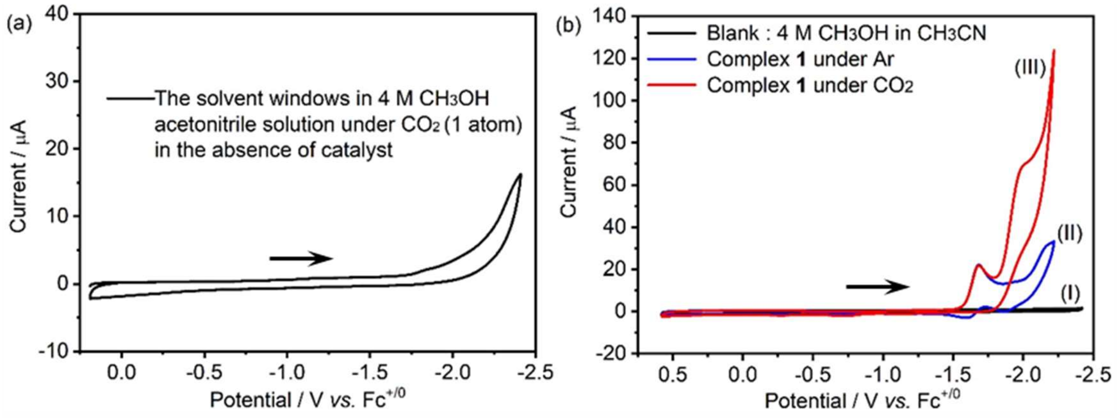

Figure S12. (a) The solvent windows in 4 M CH_3OH CH_3CN solution under CO_2. (b) Cyclic voltammogram of complex **1** (1.0 mM) in CH_3CN solution with 4 M CH_3OH at a scan rate of 100 mV/s under Ar (I) without (II) with complex **1**; (III) under CO_2.

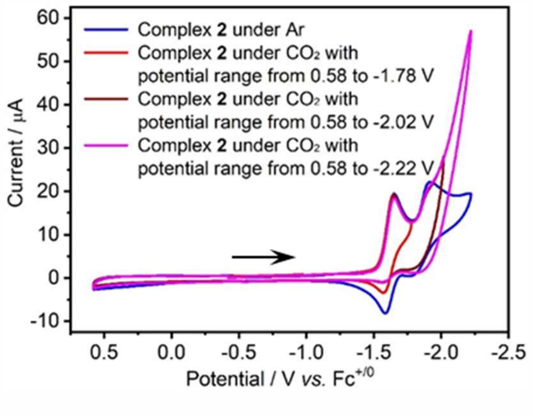

Figure S13. Cyclic voltammograms of complex **2** (1.0 mM) in acetonitrile solution (0.1 M $^{n}Bu_4NPF_6$) with varied scan range.

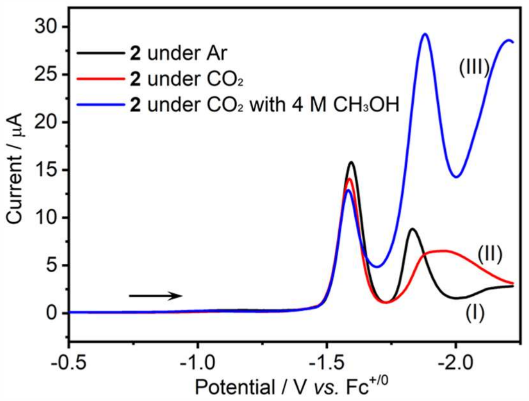

Figure S14. Differential pulse voltammograms of complex **2** (1.0 mM) in acetonitrile solution (0.1 M ^{n}Bu$_4$NPF$_6$) with a scan rate of 4 mV/s (I) under Ar or under CO_2 (b) without and (III) with 4 M methanol.

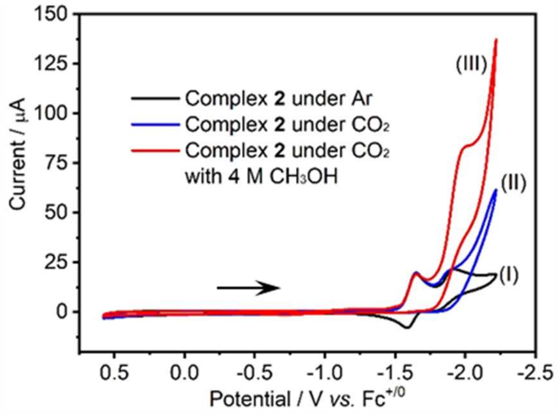

Figure S15. Cyclic voltammogram of complex **2** (1.0 mM) in acetonitrile solution (0.1 M ^{n}Bu$_4$NPF$_6$) with a scan rate of 100 mV/s (I) under Ar or under CO_2 (II) in the absence of and (III) in the presence of 4 M methanol.

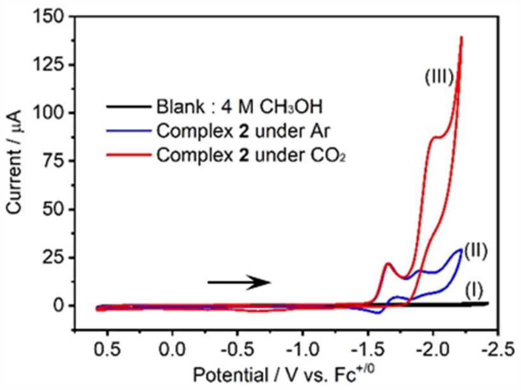

Figure S16. Cyclic voltammogram of complex **2** (1.0 mM) in acetonitrile solution (0.1 M ^{n}Bu$_4$NPF$_6$) with 4 M methanol at a scan rate of 100 mV/s. Under Ar (I) without (II) with complex **2** (1.0 mM); (III) under CO_2 (1 atm).

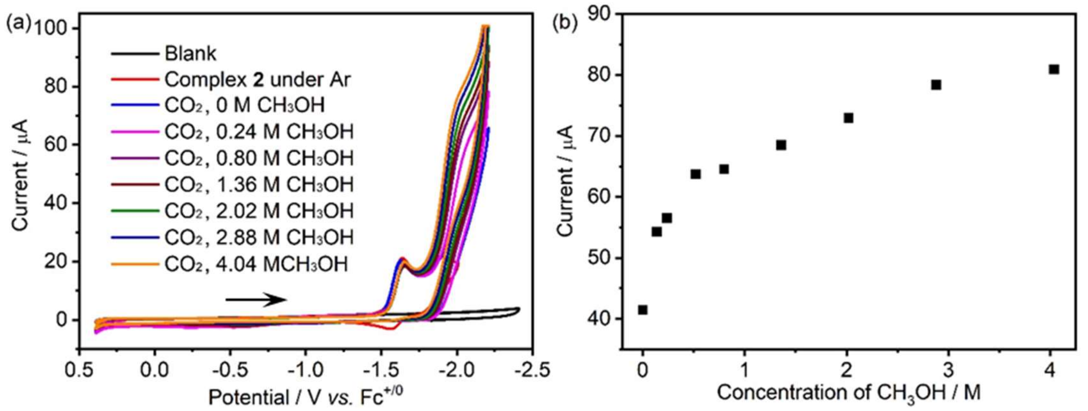

Figure S17. (a) Cyclic voltammogram of complex **2** (1.0 mM) in acetonitrile solution with varying concentration of methanol at a scan rate of 100 mV/s under CO_2; (b) The trend of catalytic peak current with increasing methanol concentration at −2 V.

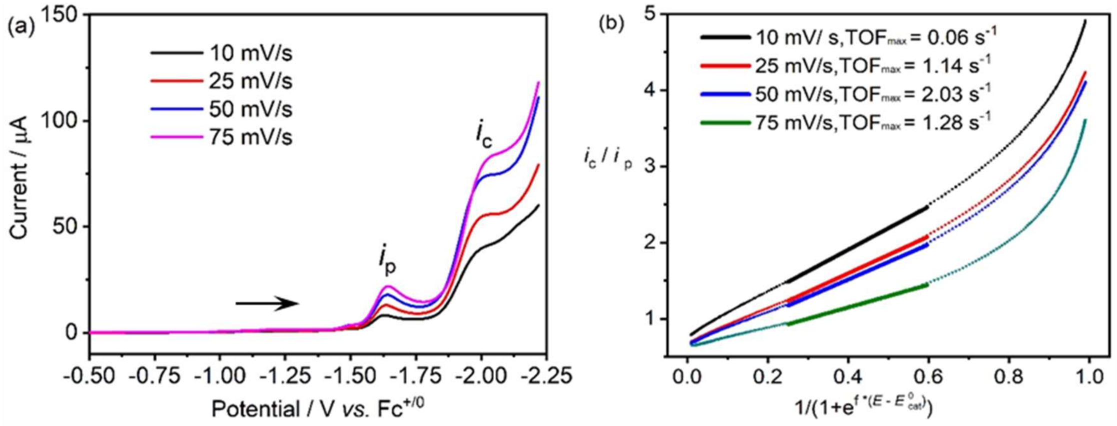

Figure S18. (a) Cyclic voltammogram of complex **1** (1 mM) with 4 M MeOH in acetonitrile solution under CO_2 with scan rate varied from 10 mV/s to 75 mV/s; (b) Foot-of-the-wave- analyses of the voltammograms with varied scan rate from 10 mV/s to 75 mV/s, respectively.

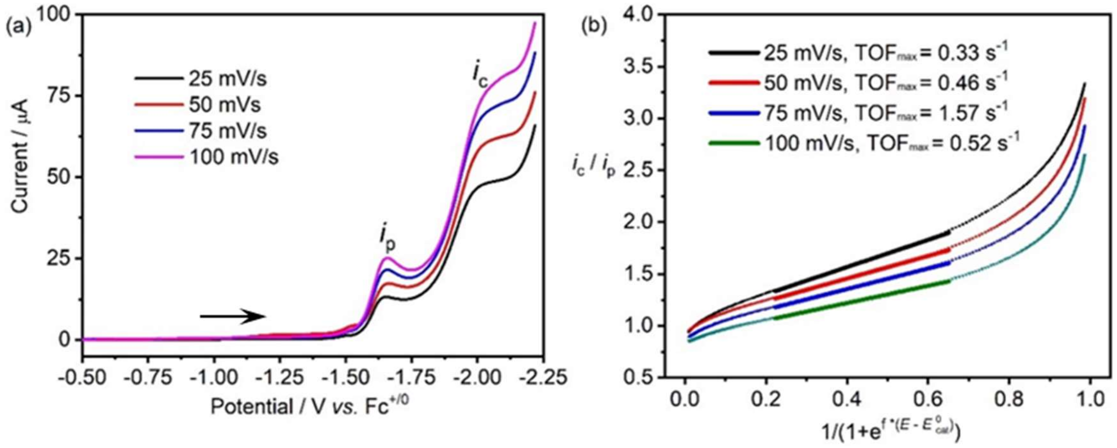

Figure S19. (a) Cyclic voltammogram of complex **2** (1 mM) with 4 M MeOH in acetonitrile solution under CO_2 with scan rate varied from 10 mV/s to 75 mV/s; (b) Foot-of-the-wave- analyses of the voltammograms with varied scan rate from 10 mV/s to 75 mV/s, respectively.

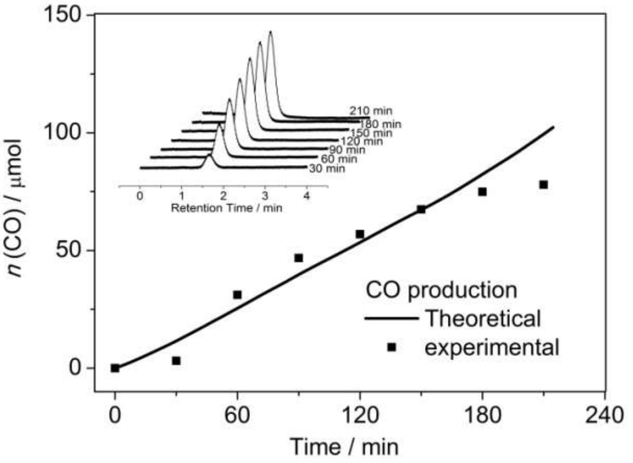

Figure S20. CO production detected by gas chromatograph (■) and in theory (line) as electrolysis time goes on. The theoretical production was transformed from electrolysis data. Inset: CO concentration detected per 30 min. CPE of complex **1** is performed at −2.1 V (*vs.* $Fc^{+/0}$). Conditions: 0.1 M $^{n}Bu_4NPF_6$; working electrode = glassy carbon; counter electrode = Pt gauze; reference electrode = Ag/AgCl.

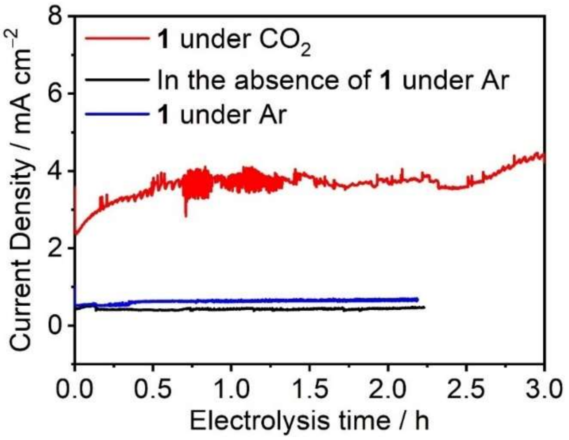

Figure S21. CPE current density over time in the absence (black line) and presence of 1 mM complex **1** under CO_2 (red line) or Ar (blue line) at −2.1 V *vs.* $Fc^{+/0}$.

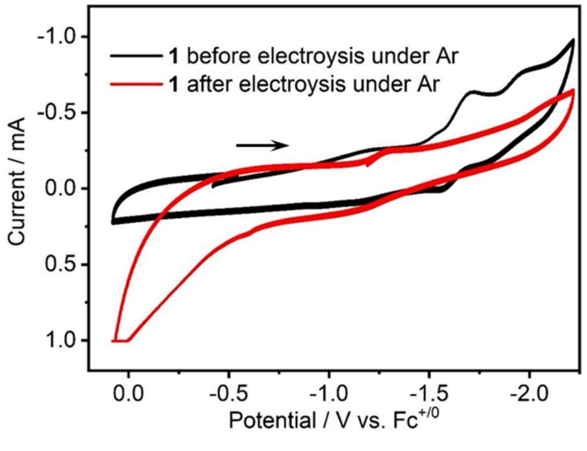

Figure S22. Cyclic voltammogram of complex **1** (1.0 mM) before and after electrolysis for 3 hours under Ar in CH_3CN solution (0.1 M $^{n}Bu_4NPF_6$) with a scan rate of 100 mV/s.

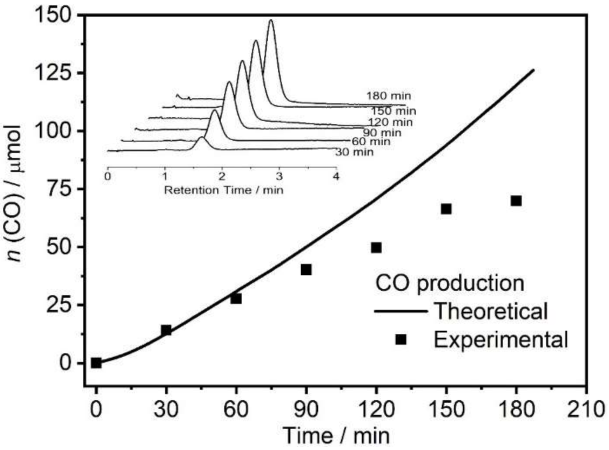

Figure S23. Gas chromatography analysis of CO production in theory (line) and experiment (■) as electrolysis time goes on. Inset: CO concentration detected per 30 min. CPE of complex **2** was run at −2.1 V *vs.* $Fc^{+/0}$. Conditions: 0.1 M $^{n}Bu_4NPF_6$; working electrode = glassy carbon; counter electrode = Pt gauze; reference electrode = Ag/AgCl.

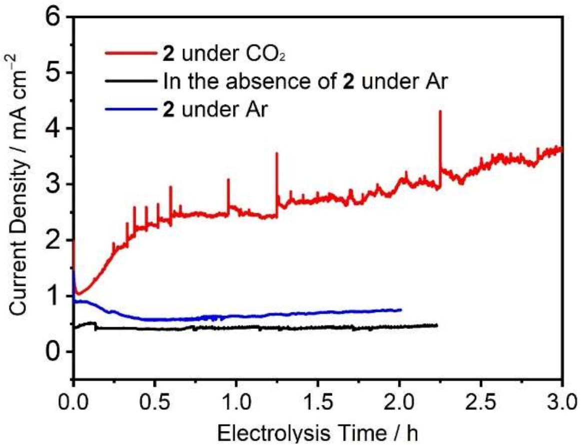

Figure S24. CPE current density over time for in the absence (black line) and presence 1 mM complex **2** under CO_2 (red line) or Ar (blue line) at −2.1 V *vs.* $Fc^{+/0}$.

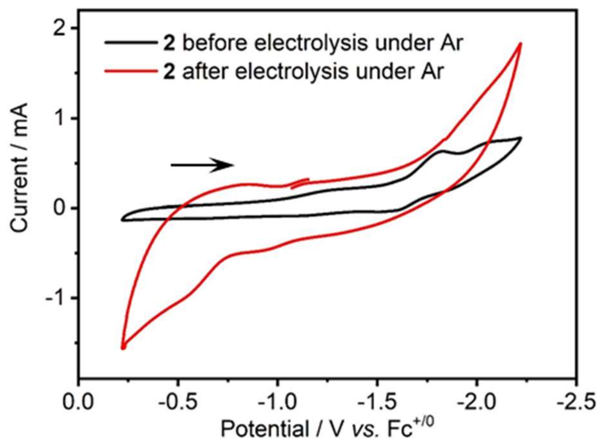

Figure S25. Cyclic voltammogram of complex **2** (1.0 mM) before and after electrolysis for 3 hours under Ar in $^{n}Bu_4NPF_6$ acetonitrile solution (0.1 M) with a scan rate of 100 mV/s.

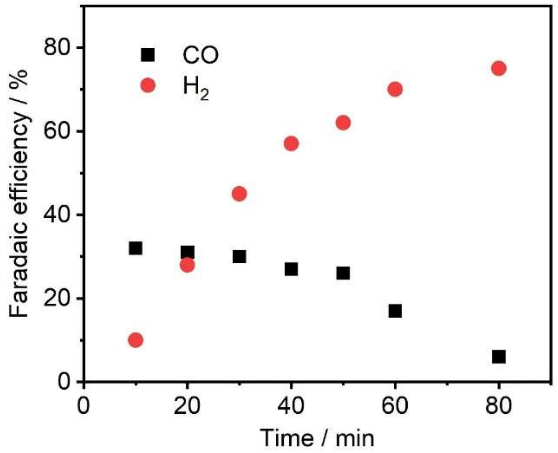

Figure S26. CO and H_2 production detected by gas chromatograph (■) and in theory (line) as electrolysis time goes on. The theoretical production was transformed from electrolysis data. Inset: CO and H_2 concentration detected per 10 min. CPE of complex **1** is performed at −2.1 V (*vs.* $Fc^{+/0}$) under CO_2 with 4 M methanol.

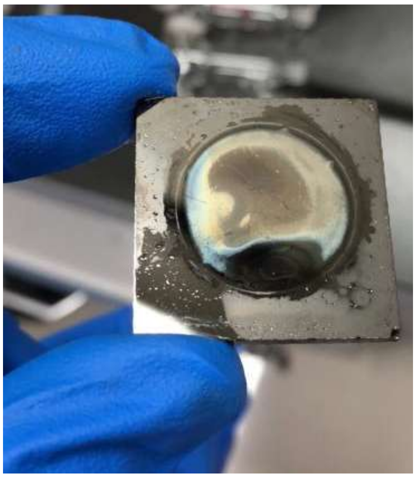

Figure S27. The photograph of GC electrode after CPE of complex **1** at −2.1 V (*vs.* $Fc^{+/0}$) under CO_2 in the presence of 4 M methanol for 80 min.

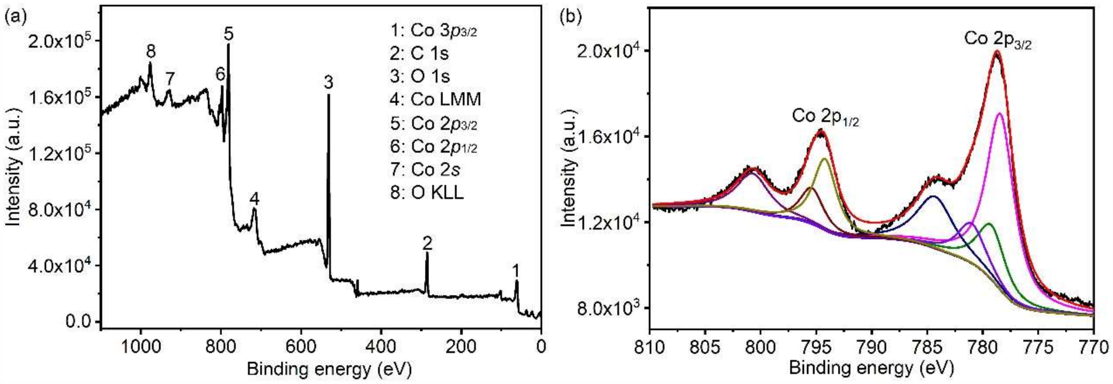

Figure S28. (a) XPS curves of the surface deposits on glassy carbon electrodes, which are formed by the deposition of complex **1** in CH_3CN solution under CO_2 with 4 M methanol at a applied potential of −2.1 V.

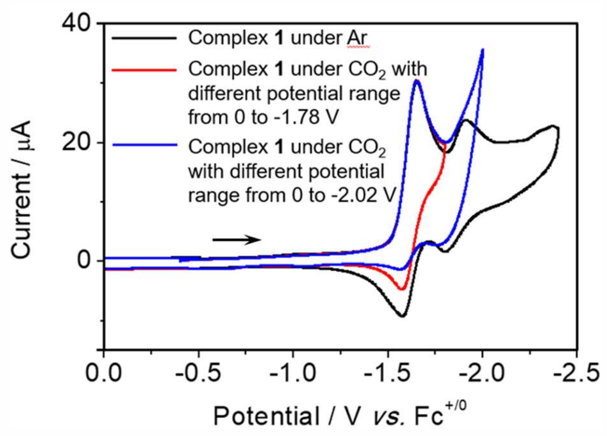

Figure S29. Cyclic voltammograms of complex **1** in CH_3CN with varied scan range.

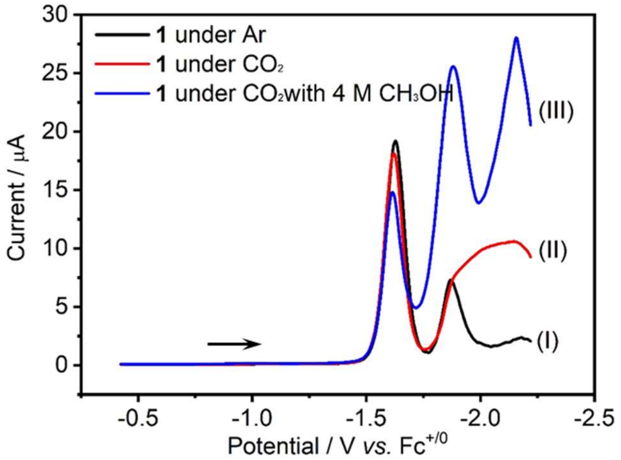

Figure S30. Differential pulse voltammograms of complex **1** (1.0 mM) in $^{n}Bu_4NPF_6$ acetonitrile solution (0.1 M) with a scan rate of 4 mV/s. (a) under Ar; under CO_2 (1 atm) (b) without and (c) with 4 M methanol.

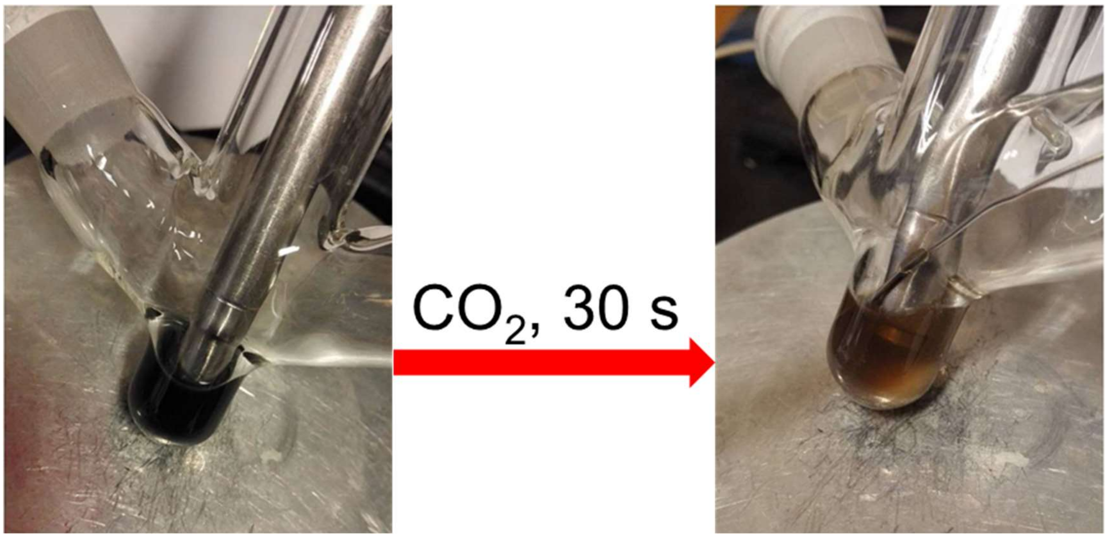

Figure S31. Freshly prepared $[Co^0(L^1)]^0$ (dark blue) solution in acetonitrile/THF (1:1) was bubbled with dry CO_2 gas for 30 s.

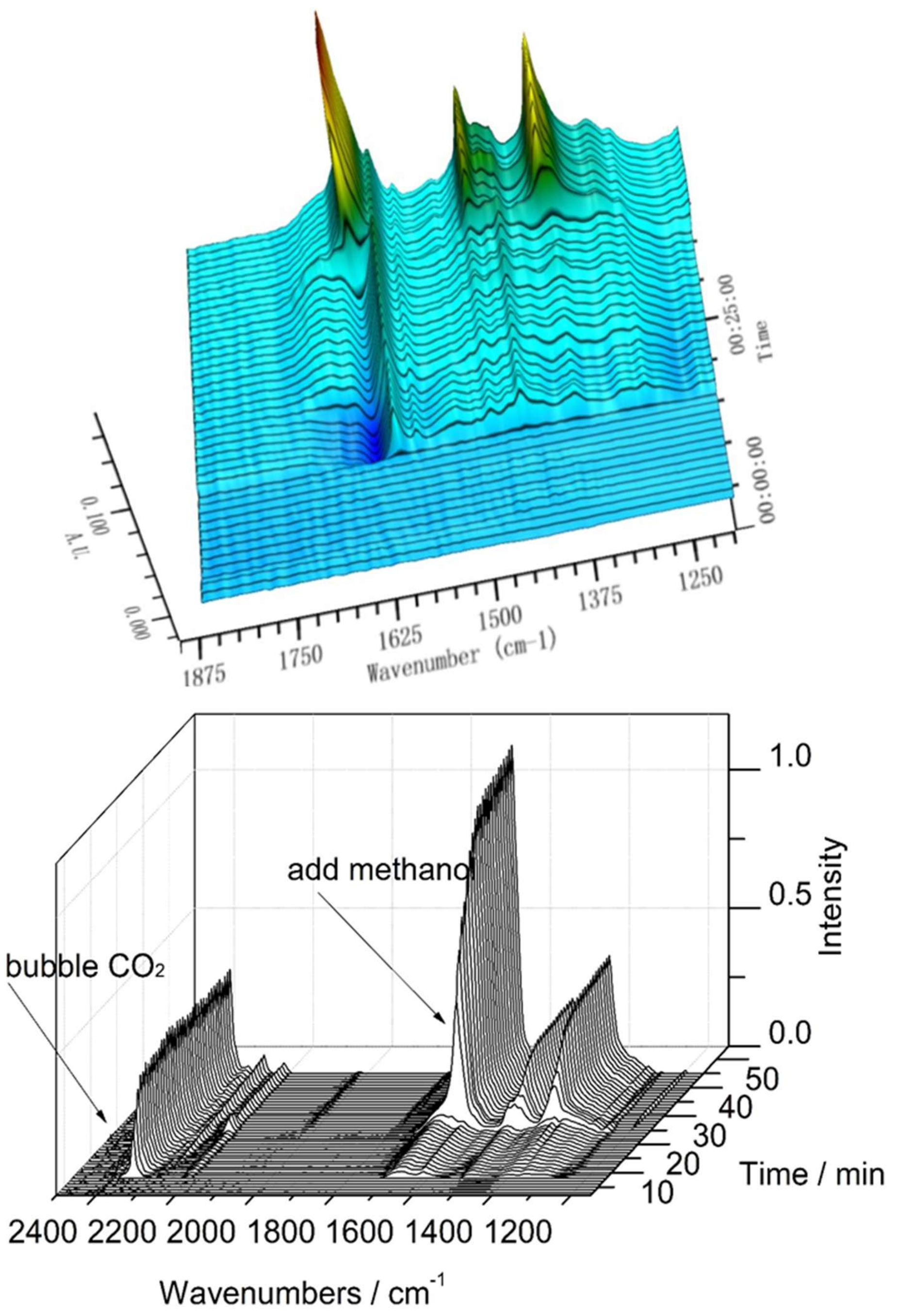

Figure S32. Freshly prepared $[Co^0(L^1)]^0$ (dark blue) solution in acetonitrile/THF (1:1) mixture was bubbled with dry CO_2 gas, then added with 2 M CH_3OH. The process was monitored by IR *in situ*. Origin drawing by METLER TOLEDO (above), handled by origin software (down).

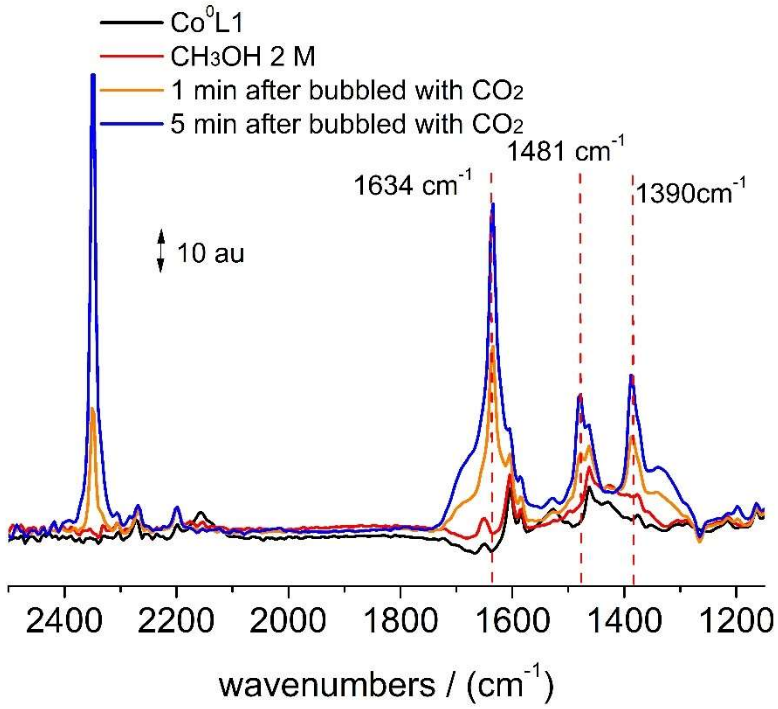

Figure S33. IR spectrum of freshly prepared $[Co^0(L^1)]^0$ (black line), which is added with 2 M methanol (red line), then bubbled with dry CO_2 (blue line). These lines were recorded by *in situ* FTIR during reaction process. The tests are performed in CH_3CN /THF (1:1) mixture under Ar with 10.0 mM $[Co^0(L^1)]^0$.

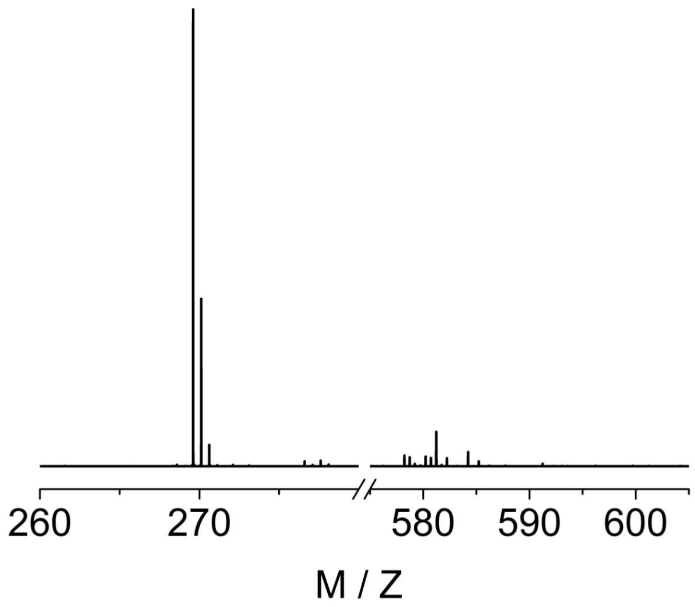

Figure S34. The full high resolution mass spectrometry (HR-MS) of CO_2 coordinated $[Co^0(L^1)]^0$ complex $[Co^{II}(L^1)-CO^{2-}]^0$.

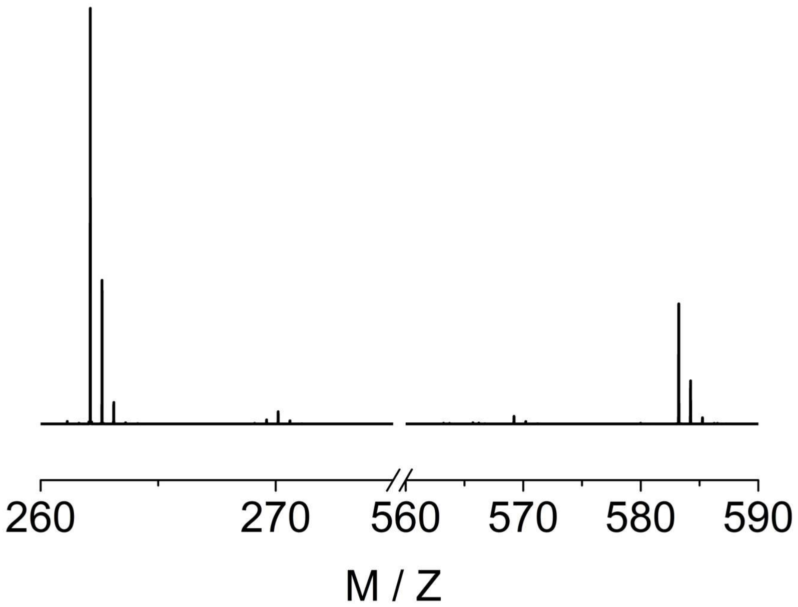

Figure S35. The full high resolution mass spectrometry (HR-MS) of CO_2 coordinated $[Co^0(L^2)]^0$ complex $[Co^{II}(L^2)-CO^{2-}]^0$.

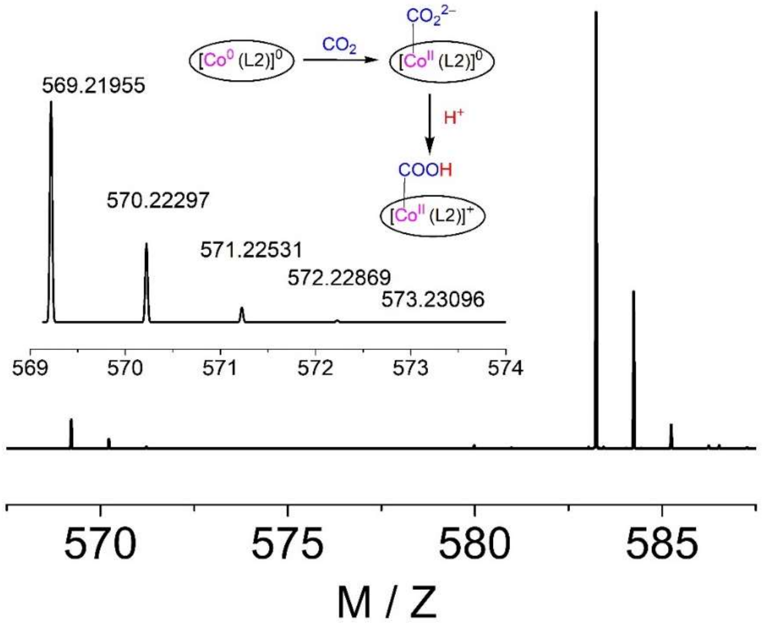

Figure S36. The observed and calculated (inset) high resolution mass spectra (HR-MS) of the protonated product of $[Co^{II}(L^2)-CO^{2-}]^0$ generated from CO_2 coordinating to $[Co^0L^2]^0$.

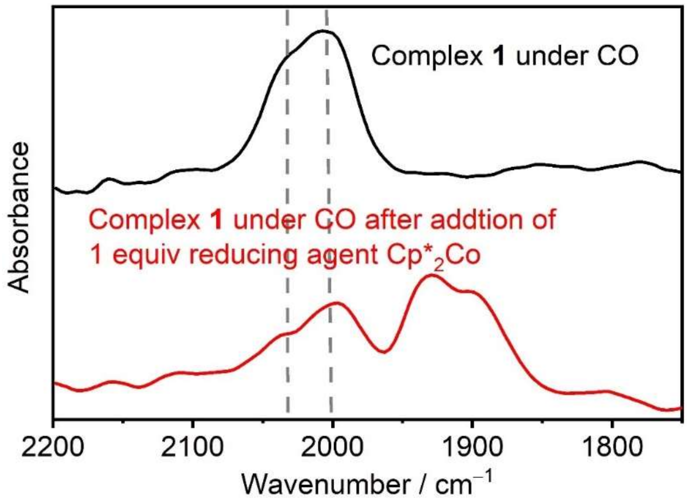

Figure S37. IR absorption spectra of complex **1** under CO atmosphere and recorded after reduction of complex **1** (6.0 mM) by 1 equiv of $Cp*_2Co$ under CO atmosphere in CH_3CN solution.

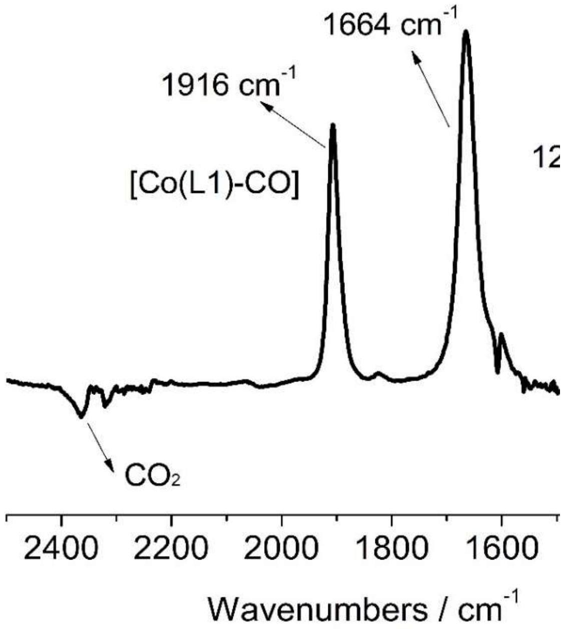

Figure S38. Differential IR-SEC spectrum of **1** (10.0 mM) recorded after electrolysis at –1.85 V (*vs.* $Fc^{+/0}$) for 10 s in CO_2 saturated CH_3CN solution in the presence of 1 M CH_3OH. The tests are performed in CH_3CN with 0.1 M $^nBu_4NPF_6$ as the supporting electrolyte.

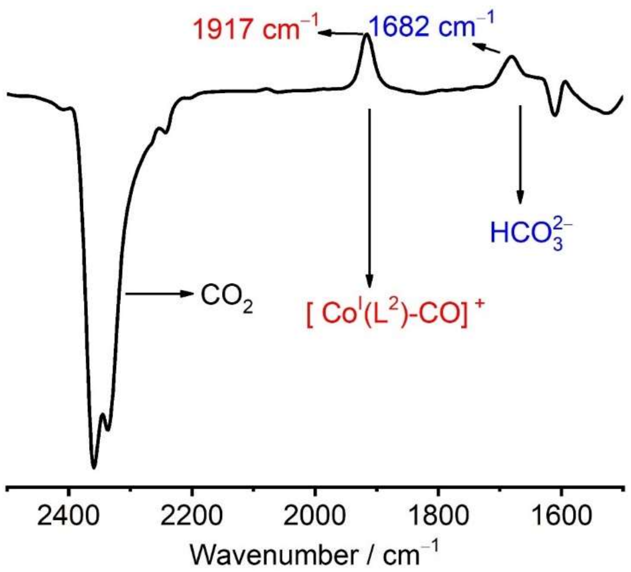

Figure S39. Differential absorbance IR spectra recorded after reduction of complex **2** (10.0 mM) at a potential of –1.85 V (vs. $Fc^{+/0}$) with varied electrolysis time in CH_3CN solution with 0.1 M $^{n}Bu_4NPF_6$ as the supporting electrolyte

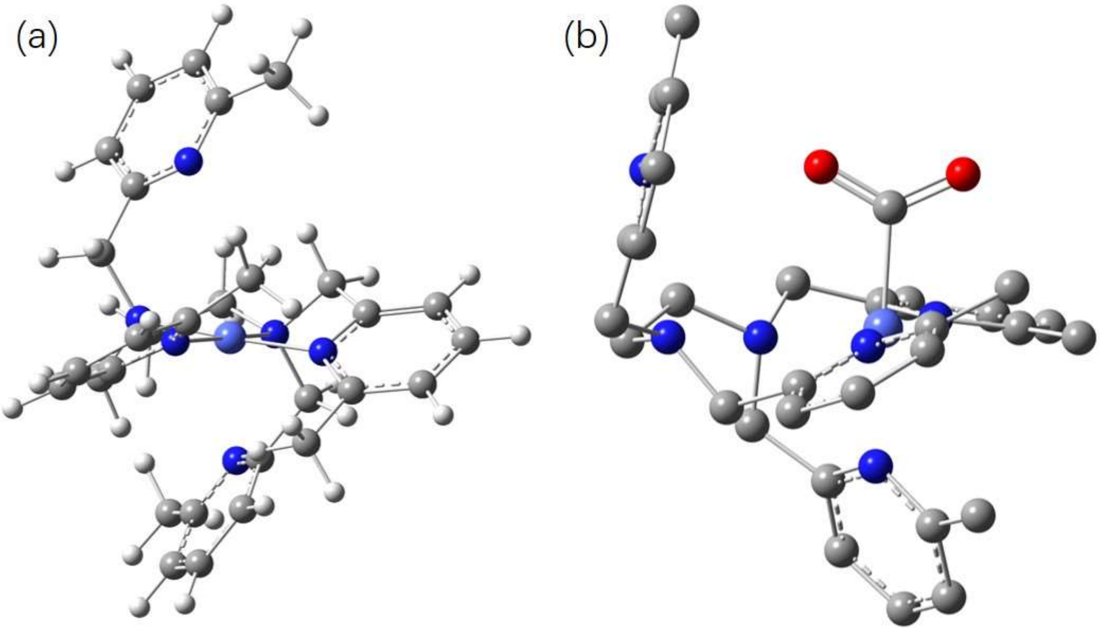

Figure S40. (a) The optimized structure of $[Co^{I}(L^{1})]^{+}$. (b) the optimized structure of $[Co^{II}(L^{1})–CO_2^{2-}]^{0}$.

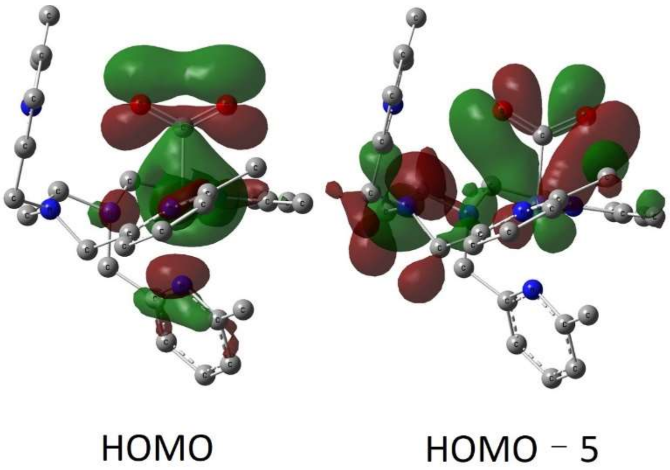

Figure 41. Calculated orbitals of $[Co^{II}(L^1)–CO_2^{2-}]^0$ generated from CO_2 coordinating to $[Co^0\ L^1]^0$: (left) the HOMO orbital where Co(d_{z^2}) forms a σ bond to the carbon atom of CO_2; (right) the HOMO–5 orbital that forms π interactions between the d_{xz} or d_{yz} orbital of Co and the p orbitals of the oxygen atoms on CO_2. The Co–C and two C–O bonds distance are 1.915, 1.270, 1.266 Å respectively, the O–C–O bond angle is 125.167°. Co (light blue), C (gray), N (blue), O (red).

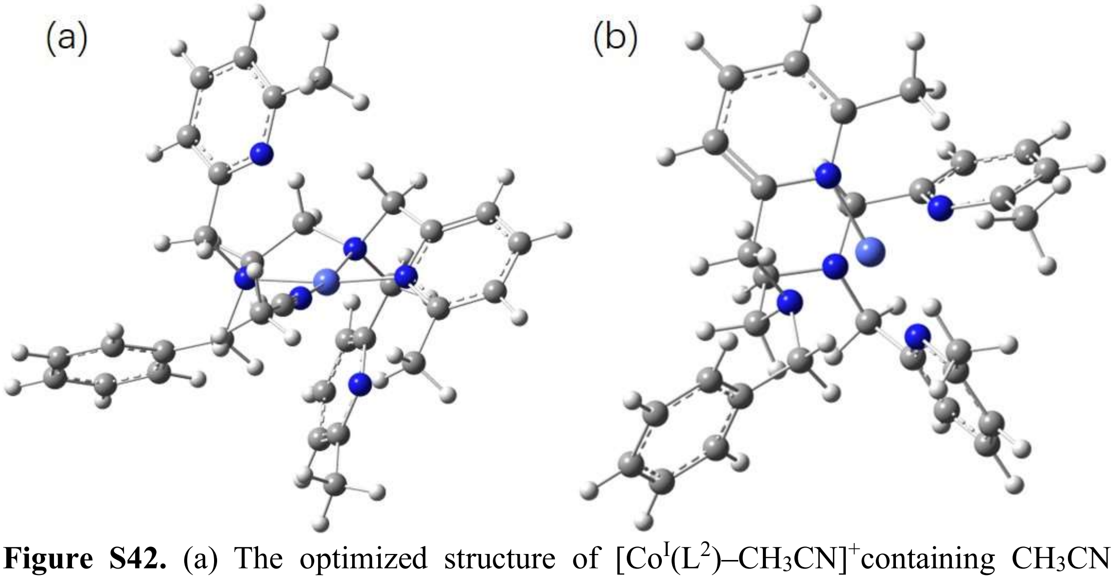

Figure S42. (a) The optimized structure of $[Co^I(L^2)–CH_3CN]^+$containing CH_3CN ligand; (b) The optimized structure of $[Co^I(L^2)]^+$ in the absence of CH_3CN ligand.

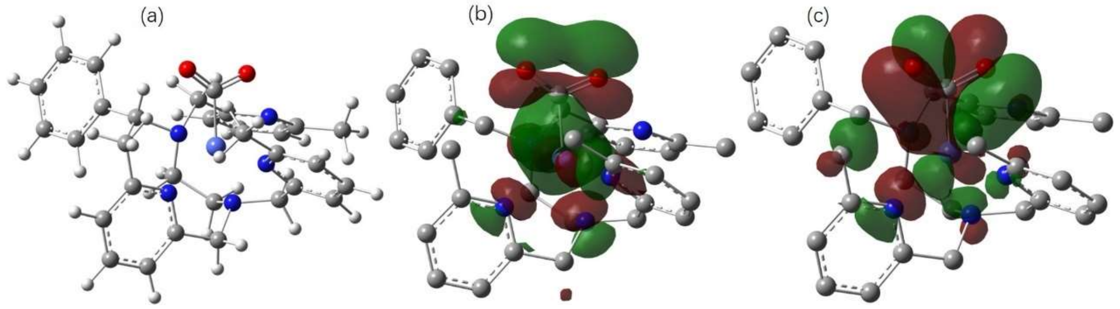

Figure S43. Calculated orbitals of $[Co^{II}(L^2)–CO_2^{2–}]^0$ generated from CO_2 coordinating to $[Co^0\ L^2]^0$: (left) the HOMO orbital where Co(d_{z^2}) forms a σ bond to the carbon atom of CO_2; (right) the HOMO–5 orbital that forms π interactions between the d_{xz} or d_{yz} orbital of Co and the p orbitals of the oxygen atoms on CO_2. The Co–C and two C–O bonds distance are 1.872, 1.283, 1.268 Å respectively, the O–C–O bond angle is 124.594°. Co (light blue), C (gray), H (white), N (blue), O (red).

Table S1. Selected Bond Lengths (Å) and Angles (deg) for **1** and **2**

Complex	**1**	**2**
Bond lengths		
Co1—N1	2.193(5)	2.179(3)
Co1—N2	2.164(5)	2.138(3)
Co1—N3	2.147(5)	2.233(3)
Co1—N4	2.186(5)	—
Co1—N5	2.292(5)	2.217(2)
Co1—N6	2.326(5)	2.279(3)
Bond angles		
N5—Co1—N6	167.87(17)	168.76(10)
N1—Co1—N2	75.22(19)	77.14(10)
N1—Co1—N3	155.15(18)	157.43(10)
N1—Co1—N4	129.29(19)	—
N2—Co1—N3	80.38(18)	81.44(10)
N4—Co1—N2	155.44(19)	—
N4—Co1—N3	75.29(18)	—
N5—Co1—N1	89.44(18)	93.12(9)
N5—Co1—N2	79.44(19)	81.28(10)
N5—Co1—N3	90.71(18)	90.57(9)
N5—Co1—N4	97.36(18)	—
N6—Co1—N1	95.06(18)	93.92(10)
N6—Co1—N2	90.81(18)	91.76(11)
N6—Co1—N3	80.48(18)	79.61(10)
N6—Co1—N4	88.51(17)	—

Table S2. Crystallographic Data and Processing Parameters for **1** and **2**

Complex	**1**	**2**
Empirical formula	$C_{31}H_{36}B_2CoF_8N_6O$	$C_{32}H_{38}B_2CoF_8N_6$
Formula weight	741.21	739.23
Crystal system	Monoclinic	Monoclinic
Space group	P2(1)/c	*Cc*
a / nm	8.5421(7)	11.2603(6)
b / nm	21.4163(17)	17.3222(10)
c / nm	18.5232(14)	17.9924(13)
β / (º)	102.653(5)	102.974(2)
V / nm^3	3.3063(5)	3.4199(4)
Z	4	4
D_c / (g · cm^{-3})	1.489	1.436
μ / mm^{-1}	0.601	0.578
T (K)	296(2)	296(2)
F(000)	1524	1524
Crystal size / mm^3	0.23 × 0.21 × 0.18	0.31 × 0.25× 0.21
θ range / (°)	$2.07 < \theta < 25.00$	$2.24 < \theta < 25.00$
Reflections collected	18587	43104
Unique reflections	13308	5719
R_{int}	0.0866	0.0207
GOF	1.029	1.042
Final R indices	$R1$ = 0.0789,	$R1$ = 0.0412,
[I>2σ(I)]	$wR2$ = 0.2177	$wR2$ = 0.1140
R indices (all data)	$R1$ = 0.1301,	$R1$ = 0.0424,
	$wR2$ = 0.2394	$wR2$ = 0.1140

Table S3. Redox Potentials ($E_{1/2}$) of Complexes **1** and **2**.

Complex	$Co^{III/II}$	$Co^{II/I}$	$Co^{I/0}$	ΔE [a]
**1**	—	–1.66	–1.89	0.23
**2**	—	–1.64	–1.87	0.23

Table S4. Electrochemical Studies of **1** and **2**

Complex	i_{cat}/i_p	TOF_{cv} (s^{-1})	TON_{total}	$TOF_{average}$ (h^{-1})
**1**	5.5	2.59	9.5	2.03
**2**	5.2	1.57.	8.8	1.57

Geometry optimized xyz coordinates for [Co^{I}(L^{1})]$^{+}$ from Gaussian calculations.

Co	0.195533	-0.46386	0.069544
N	-4.01194	1.18325	-0.80304
N	-0.76307	0.584028	-1.29663
N	0.358485	-1.73839	-1.33257
N	2.728255	1.931743	-0.00236
C	-3.23542	0.087863	-0.83194
C	-2.09225	0.104705	-1.82365
H	-1.93855	-0.90374	-2.20978
H	-2.37473	0.754593	-2.66119
N	-0.05355	1.028029	1.332709
C	-0.86046	1.985674	-0.78354
H	-1.68051	2.526362	-1.26818
H	0.069008	2.503177	-1.02188
C	0.249224	0.41894	-2.38935
H	1.151954	0.957988	-2.08998
H	-0.09797	0.840517	-3.34355
C	0.508199	-1.06536	-2.51473
C	-1.07517	1.935766	0.728553
H	-1.02463	2.940956	1.164099
H	-2.0631	1.535176	0.953979
C	1.904791	2.61143	0.814227
C	1.134926	1.80387	1.837862
H	1.820026	1.084029	2.287627
H	0.779553	2.473093	2.632481
C	0.34375	-3.09806	-1.37127
C	-5.05782	1.220589	0.044445
C	-0.58595	0.215691	2.47515
H	-1.56766	-0.16883	2.185045
C	1.799804	4.005489	0.781357
H	1.140191	4.524602	1.469557
C	-3.48098	-1.03225	-0.02877
H	-2.84278	-1.90689	-0.09888
C	3.469167	2.608999	-0.8968
C	-5.35946	0.147107	0.891744
H	-6.21026	0.206214	1.56337
C	-4.56231	-0.99563	0.849511
H	-4.78505	-1.84693	1.486425
C	3.406429	4.006192	-1.00163
H	4.0171	4.523961	-1.73461
C	2.562745	4.711476	-0.14894
H	2.502223	5.794565	-0.20297
C	0.644448	-3.78607	-2.55221
H	0.642449	-4.87162	-2.53974

C	0.79458	-1.69699	-3.71277
H	0.906066	-1.11171	-4.61979
C	0.909721	-3.09042	-3.72622
H	1.146779	-3.61902	-4.64407
H	-0.71584	0.817664	3.385857
C	0.4009	-0.90531	2.703918
C	0.69064	-1.42973	3.951929
C	2.102592	-2.1391	1.681966
C	1.699032	-2.3922	4.057386
H	0.151023	-1.07811	4.825285
H	1.944526	-2.83667	5.016671
C	2.425927	-2.71309	2.916746
H	3.270589	-3.39285	2.971539
N	1.036907	-1.30201	1.559277
C	4.387977	1.792925	-1.77166
H	5.148931	1.290499	-1.16291
H	3.829556	1.011289	-2.29878
H	4.896231	2.416709	-2.51177
C	-0.0715	-3.84971	-0.13376
H	-0.55042	-3.17006	0.572734
H	-0.77938	-4.64117	-0.40182
H	0.772895	-4.33257	0.370367
C	-5.89458	2.475401	0.024777
H	-5.26822	3.358386	0.193312
H	-6.3731	2.602259	-0.95365
H	-6.6759	2.449869	0.789111
C	2.973413	-2.38448	0.477688
H	4.026925	-2.36869	0.775388
H	2.786331	-3.35853	0.012783
H	2.806621	-1.60951	-0.2717

Geometry optimized xyz coordinates for $[Co^{II}(L^1)–CO_2^{2–}]^0$ from Gaussian calculations.

Symbolic Z-matrix:

Charge = 0 Multiplicity = 2

Co	-0.82188	-0.07648	0.51442
N	4.1329	-1.41291	-0.51687
N	1.94615	-0.10432	-1.95055
N	0.75515	1.32239	0.69139
N	-2.04061	1.20125	-0.86916
C	4.30327	-0.21426	-1.09285
C	3.36579	0.12281	-2.24604
H	3.54156	1.16168	-2.56065
H	3.65671	-0.50081	-3.10081
N	-0.93341	-1.38053	-1.47552
C	1.25989	-1.044	-2.83415
H	2.00759	-1.71887	-3.26183
H	0.7946	-0.53291	-3.69702
C	1.17316	1.11936	-1.74102
H	0.11883	0.86775	-1.77953
H	1.36621	1.85857	-2.5399
C	1.42351	1.78172	-0.40759
C	0.27092	-1.97255	-2.12566
H	-0.05337	-2.72617	-2.86544
H	0.80862	-2.4787	-1.32277
C	-2.59068	0.4818	-1.87406
C	-1.80231	-0.68403	-2.44138
H	-2.48652	-1.38471	-2.94194
H	-1.17505	-0.26654	-3.23604
C	1.02228	1.94699	1.87675
C	4.9364	-1.7725	0.49871
C	-1.66065	-2.51834	-0.87548
H	-0.9412	-3.04065	-0.23755
C	-3.78382	0.84753	-2.49881
H	-4.19492	0.22105	-3.28411
C	5.31201	0.67046	-0.69656
H	5.42213	1.6336	-1.18604
C	-2.61914	2.38528	-0.54467
C	5.9709	-0.94425	0.95436
H	6.60662	-1.26457	1.77433
C	6.16241	0.29203	0.34105
H	6.95548	0.95606	0.67456
C	-3.81015	2.81232	-1.1395
H	-4.24727	3.75849	-0.83824

C	-4.41759	2.02311	-2.11152
H	-5.35182	2.32961	-2.57262
C	1.94007	3.00149	1.9743
H	2.1119	3.45113	2.94652
C	2.3164	2.85113	-0.36248
H	2.78799	3.18268	-1.28145
C	2.59374	3.47491	0.84832
H	3.29412	4.30253	0.90908
H	-2.00928	-3.21597	-1.65315
C	-2.81168	-2.08873	-0.01375
C	-4.01511	-2.78883	-0.00613
C	-3.50277	-0.71577	1.73005
C	-4.99847	-2.42775	0.91105
H	-4.16473	-3.60755	-0.70232
H	-5.9487	-2.95224	0.94445
C	-4.72705	-1.38831	1.79422
H	-5.45507	-1.08716	2.54033
N	-2.56003	-1.0481	0.81339
C	-1.91931	3.27651	0.44781
H	-0.92474	3.54855	0.07999
H	-2.48927	4.19495	0.60912
H	-1.78142	2.78783	1.41498
C	0.2837	1.55466	3.12487
H	0.11947	0.47589	3.18653
H	0.83434	1.89655	4.00638
H	-0.6985	2.04361	3.15638
C	4.65359	-3.11388	1.13042
H	3.61829	-3.14656	1.48998
H	4.76582	-3.91965	0.39509
H	5.32577	-3.3158	1.96925
C	-3.17411	0.38046	2.69874
H	-3.90747	0.4204	3.50875
H	-2.18135	0.18868	3.11291
H	-3.16798	1.35815	2.20594
C	-0.01669	-1.40004	1.64139
O	-0.3644	-1.42538	2.86223
O	0.77706	-2.2189	1.09231

Geometry optimized xyz coordinates for $[Co^{I}(L^{2})–CH_3CN]^{+}$ from Gaussian calculations

Symbolic Z-matrix:

Charge = 1 Multiplicity = 1

Co	0.80199	-0.31885	0.45958
N	-1.15249	-3.01651	-0.47544
N	0.99894	0.27323	2.15615
N	0.83445	-0.69461	-1.48611
N	2.37056	-1.41472	0.48942
N	1.75354	2.87434	-0.19079
C	-0.90262	-2.57869	-1.72123
C	0.49639	-2.07133	-1.99749
H	1.20271	-2.76256	-1.53417
H	0.67891	-2.07883	-3.08001
N	-0.79093	0.87813	0.12295
C	0.01863	0.36006	-2.1578
H	-0.28403	0.04696	-3.16481
H	0.63759	1.24955	-2.2705
C	2.29953	-0.48053	-1.71406
H	2.50176	0.58346	-1.5681
H	2.59803	-0.74733	-2.73708
C	3.04346	-1.29671	-0.69203
C	-1.20251	0.66082	-1.30104
H	-1.75372	1.52162	-1.69653
H	-1.8805	-0.19093	-1.32723
C	0.45103	3.0613	-0.46376
C	-0.55716	2.33867	0.40834
H	-0.22586	2.40128	1.44604
H	-1.51535	2.86133	0.32257
C	2.90467	-2.21801	1.44884
C	-2.36185	-3.53122	-0.19259
C	-1.87225	0.37214	1.05965
H	-1.48727	0.519	2.0698
H	-1.92921	-0.70367	0.88259
C	0.01626	3.93788	-1.46443
H	-1.04407	4.08639	-1.64359
C	-1.85105	-2.65083	-2.74626
H	-1.60764	-2.30783	-3.74693
C	2.67665	3.5355	-0.91158
C	-3.37031	-3.62471	-1.16333
H	-4.33519	-4.04986	-0.90552
C	-3.10904	-3.1793	-2.45605
H	-3.86896	-3.24967	-3.22885

C	2.31912	4.4121	-1.94582
H	3.08822	4.92807	-2.51205
C	0.9704	4.61712	-2.22155
H	0.66497	5.29957	-3.00943
C	4.11965	3.30012	-0.53923
H	4.3123	3.64839	0.48242
H	4.80018	3.82496	-1.2151
H	4.35811	2.2308	-0.56468
C	-3.2539	0.99173	0.94663
C	2.11038	-2.50244	2.69544
H	1.04334	-2.37166	2.50815
H	2.39446	-1.83755	3.51873
H	2.29356	-3.52908	3.02732
C	4.15819	-2.81405	1.26969
H	4.55615	-3.43894	2.06291
C	1.16989	0.7208	3.21834
C	4.27733	-1.87976	-0.92912
H	4.75948	-1.75131	-1.89265
C	-2.58023	-4.00968	1.22102
H	-2.53488	-3.16965	1.92465
H	-1.79553	-4.71671	1.51126
H	-3.55149	-4.49866	1.33438
C	-4.23156	0.43508	0.10741
H	-4.00261	-0.455	-0.47268
C	-3.60633	2.10685	1.72395
H	-2.87843	2.53367	2.40947
C	4.86713	-2.63278	0.08803
H	5.83837	-3.09592	-0.05445
C	-5.83649	2.11425	0.78676
H	-6.83142	2.5464	0.7248
C	-4.88217	2.6674	1.64344
H	-5.13186	3.52985	2.25536
C	-5.50904	0.99193	0.02299
H	-6.25029	0.54246	-0.63213
C	1.40174	1.28371	4.54173
H	2.07027	2.14901	4.47988
H	1.8592	0.53433	5.19624
H	0.45472	1.60332	4.98843

Geometry optimized xyz coordinates for $[Co^{I}(L^{2})]^{+}$ **from Gaussian calculations**

Symbolic Z-matrix:

Charge = 1 Multiplicity = 1

Co	-0.53403	0.17436	-0.25452
N	-0.31322	-1.87313	-0.19201
N	-0.72802	-0.0801	1.89687
N	-2.50664	-0.14573	-0.13562
N	-0.61657	2.07114	-0.56263
C	-0.5442	-2.42683	1.02287
C	-0.76954	-1.51321	2.22021
H	-1.73828	-1.7612	2.66836
H	-0.0213	-1.75052	2.98732
N	1.48647	0.41767	0.10543
C	0.46017	0.62329	2.39561
H	0.64998	0.41898	3.46008
H	0.29204	1.69779	2.29431
C	-2.00681	0.60134	2.12378
H	-1.85055	1.67552	1.9786
H	-2.39336	0.46027	3.14356
C	-3.0041	0.09247	1.1107
C	1.67307	0.19524	1.57939
H	2.57134	0.71104	1.93638
H	1.84084	-0.87508	1.72428
C	0.54397	2.68393	-0.12098
C	1.75847	1.83227	-0.27056
H	2.01652	1.82422	-1.33718
H	2.6175	2.23708	0.27544
C	-3.35262	-0.64767	-1.06947
C	-0.06536	-2.70753	-1.23873
C	2.44373	-0.47653	-0.64859
H	2.2309	-0.3243	-1.7075
H	2.17364	-1.5023	-0.39889
C	0.63362	3.9998	0.29641
H	1.58814	4.37581	0.65273
C	-0.56984	-3.80441	1.23125
H	-0.75986	-4.19389	2.22668
C	-1.68201	2.93719	-0.76013
C	-0.09087	-4.09688	-1.08942
H	0.10235	-4.72475	-1.95326
C	-0.35142	-4.65742	0.15552
H	-0.37454	-5.73494	0.28676
C	-1.63321	4.27141	-0.35377
H	-2.51054	4.88782	-0.52773
C	-0.49236	4.82336	0.22536

H	-0.46379	5.85787	0.55088
C	-2.89247	2.48067	-1.53028
H	-2.60573	1.77933	-2.31848
H	-3.36985	3.34486	-2.00275
H	-3.6509	1.99639	-0.90831
C	3.92664	-0.26583	-0.39039
C	-2.83371	-0.94504	-2.44668
H	-1.94549	-0.35088	-2.66547
H	-3.60138	-0.74352	-3.1999
H	-2.56256	-2.00331	-2.53147
C	-4.70132	-0.8915	-0.7801
H	-5.34136	-1.28801	-1.56131
C	-4.33217	-0.12792	1.45144
H	-4.66754	0.07743	2.46248
C	0.28441	-2.12201	-2.58219
H	0.18173	-1.03511	-2.56016
H	-0.36051	-2.52675	-3.36865
H	1.31618	-2.37738	-2.85069
C	4.59375	-0.99175	0.60876
H	4.04366	-1.71015	1.2113
C	4.67791	0.61709	-1.18162
H	4.19434	1.16316	-1.98735
C	-5.20542	-0.62281	0.48409
H	-6.24965	-0.80508	0.71815
C	6.69056	0.07782	0.04844
H	7.75568	0.20992	0.21724
C	6.04548	0.79247	-0.9632
H	6.6072	1.48	-1.58955
C	5.96115	-0.81991	0.83108
H	6.45685	-1.39388	1.60917

Geometry optimized xyz coordinates for $[Co^{II}(L^2)–CO_2^{2–}]^0$ from Gaussian calculations.

Symbolic Z-matrix:

Charge = 0 Multiplicity = 2

Co	-0.90469	-0.2216	-0.45642
N	-1.06651	-2.19521	0.69918
N	-1.07819	0.4994	1.48964
N	-2.88228	0.20037	-0.46448
N	0.77989	3.36168	-0.28881
C	-1.25716	-1.92977	2.01342
C	-1.70409	-0.52672	2.35155
H	-2.78615	-0.48317	2.18776
H	-1.53516	-0.30858	3.41434
N	1.18618	0.0016	-0.17686
C	0.26788	0.9061	1.96922
H	0.345	0.80094	3.05958
H	0.40535	1.96031	1.7394
C	-1.98929	1.64736	1.27333
H	-1.41453	2.42384	0.75777
H	-2.35814	2.07366	2.21765
C	-3.14779	1.21319	0.40219
C	1.35629	0.06276	1.3043
H	2.34469	0.43808	1.59523
H	1.28266	-0.96245	1.6728
C	1.83882	2.53425	-0.21153
C	1.74618	1.20081	-0.92693
H	1.11458	1.31876	-1.80839
H	2.76288	0.95111	-1.2397
C	-3.90519	-0.28413	-1.21548
C	-0.81177	-3.47403	0.33765
C	1.8357	-1.24771	-0.72661
H	1.53354	-1.30813	-1.77308
H	1.35536	-2.07975	-0.21095
C	3.02618	2.91042	0.42971
H	3.87217	2.23068	0.45492
C	-1.14213	-2.89305	3.01087
H	-1.28813	-2.61952	4.05097
C	0.85615	4.58396	0.26796
C	-0.68609	-4.49418	1.29388
H	-0.46786	-5.5049	0.96293
C	-0.8406	-4.20445	2.64245
H	-0.7353	-4.9812	3.39425
C	2.00767	5.02347	0.93468

H	2.03854	6.01709	1.37093
C	3.10591	4.17174	1.01687
H	4.01471	4.48953	1.52012
C	-0.36663	5.45967	0.14729
H	-0.7315	5.46909	-0.88492
H	-0.15698	6.48717	0.45722
H	-1.18079	5.07603	0.77489
C	3.34374	-1.38074	-0.60136
C	-3.67909	-1.52539	-2.03278
H	-3.59855	-2.39287	-1.36457
H	-2.75019	-1.48442	-2.60409
H	-4.52196	-1.69932	-2.70766
C	-5.17244	0.31034	-1.18034
H	-5.95993	-0.09175	-1.80922
C	-4.39244	1.82523	0.49756
H	-4.54695	2.62815	1.21112
C	-0.69976	-3.80735	-1.12543
H	-0.57798	-2.91632	-1.75041
H	-1.61269	-4.32607	-1.45057
H	0.13246	-4.49949	-1.29978
C	3.94431	-1.84905	0.57856
H	3.32315	-2.11986	1.42815
C	4.17708	-1.08952	-1.69343
H	3.73215	-0.75864	-2.62901
C	-5.42094	1.3836	-0.33227
H	-6.4019	1.8478	-0.29665
C	6.14479	-1.67945	-0.41635
H	7.22288	-1.79432	-0.344
C	5.56349	-1.23209	-1.60434
H	6.18631	-1.0005	-2.46422
C	5.3296	-1.99268	0.67432
H	5.7711	-2.3585	1.59751
C	-0.73198	-0.33654	-2.31782
O	-0.83134	0.8882	-2.68692
O	-0.48591	-1.30451	-3.09864
